# Genetic Dissection of Extreme Seed-Flooding Tolerance in a Wild Soybean PI342618B by Linkage Mapping and Candidate Gene Analysis

**DOI:** 10.3390/plants12122266

**Published:** 2023-06-10

**Authors:** Zhe-Ping Yu, Wen-Huan Lv, Ripa Akter Sharmin, Jie-Jie Kong, Tuan-Jie Zhao

**Affiliations:** 1 National Center for Soybean Improvement, Key Laboratory of Biology and Genetics and Breeding for Soybean, Ministry of Agriculture, State Key Laboratory of Crop Genetics and Germplasm Enhancement, Nanjing Agricultural University, Nanjing 210095, China; 2 Institute of Horticulture, Zhejiang Academy of Agricultural Sciences, Hangzhou 310021, China; 3 Department of Botany, Jagannath University, Dhaka 1100, Bangladesh

**Keywords:** soybean, seed-flooding tolerance, quantitative trait loci, ERF transcription factor

## Abstract

Seed-flooding stress is one of major abiotic constraints that adversely affects soybean production worldwide. Identifying tolerant germplasms and revealing the genetic basis of seed-flooding tolerance are imperative goals for soybean breeding. In the present study, high-density linkage maps of two inter-specific recombinant inbred line (RIL) populations, named NJIRNP and NJIR4P, were utilized to identify major quantitative trait loci (QTLs) for seed-flooding tolerance using three parameters viz., germination rate (GR), normal seedling rate (NSR), and electrical conductivity (EC). A total of 25 and 18 QTLs were detected by composite interval mapping (CIM) and mixed-model-based composite interval mapping (MCIM), respectively, and 12 common QTLs were identified through both methods. All favorable alleles for the tolerance are notably from the wild soybean parent. Moreover, four digenic epistatic QTL pairs were identified, and three of them showed no main effects. In addition, the pigmented soybean genotypes exhibited high seed-flooding tolerance compared with yellow seed coat genotypes in both populations. Moreover, out of five identified QTLs, one major region containing multiple QTLs associated with all three traits was identified on Chromosome 8, and most of the QTLs within this hotspot were major loci (*R*^2^ > 10) and detectable in both populations and multiple environments. Based on the gene expression and functional annotation information, 10 candidate genes from QTL “hotspot 8-2” were screened for further analysis. Furthermore, the results of qRT-PCR and sequence analysis revealed that only one gene, *GmDREB2* (*Glyma.08G137600*), was significantly induced under flooding stress and displayed a TTC tribasic insertion mutation of the nucleotide sequence in the tolerant wild parent (PI342618B). *GmDREB2* encodes an ERF transcription factor, and the subcellular localization analysis using green fluorescent protein (GFP) revealed that *GmDREB2* protein was localized in the nucleus and plasma membrane. Furthermore, overexpression of *GmDREB2* significantly promoted the growth of soybean hairy roots, which might indicate its critical role in seed-flooding stress. Thus, *GmDREB2* was considered as the most possible candidate gene for seed-flooding tolerance.

## 1. Introduction

Natural disasters are becoming more often as the world’s climate changes, resulting in a significant drop in crop production. Among these various abiotic stresses, flooding stress has emerged as the greatest threat to crop growth and development, affecting crop production and yield worldwide [1]. Due to excessive rainfall and poor drainage, the soil becomes flooded, causing hypoxia in below-ground parts and inhibiting plant growth [2]. Soybeans (*Glycine max* [L.] Merr.) are one of the most important commercial crops in the world due to its high nutritional values, containing protein, oil, carbohydrates, and isoflavones [3]. However, soybeans are relatively susceptible to flooding stress, with considerable growth and yield inhibition under flood conditions [4,5]. Flooding stress affects soybeans at different growth and development stages, including germination, vegetative, and reproductive stages, which results in a great loss in soybean yields [3]. Moreover, flooding imposes a severe selection pressure on plants since excess water in the living surroundings can deprive them of oxygen, carbon dioxide, and light [6]. Flooding stress is considered as a primary limiting factor in soybean cultivation because 60–70% of annual precipitation occurs during the growth and development period [7]. Additionally, several studies have displayed that flooding stress has a consistent impact on soybean growth and grain yields around the world, particularly in key soybean-producing countries such as China and United States [8]. For example, in the Mississippi Delta region, flooding stress reduced overall soybean yield by up to 25% [9]. Previous studies also focused on the understanding of flooding influences on different soybean growth stages and yield cutbacks. It is reported that flooding reduced soybean yields by 17–43% at the vegetative growth stage and 50–56% at the reproductive stage [8,10]. Similarly, Sullivan et al. (2001) exhibited a 20% yield loss when soybean plots were flooded for three days at V2 and V3 growth stages [11].

Soybean flooding tolerance is considered to be a complex quantitative trait. Hence, QTL mapping is an effective way to unravel the underlying genetic mechanism of the tolerance. 27 QTLs on Soybase (http://www.soybase.org) (accessed on 5 February 2023) have been reported to be linked to flooding tolerance at seedling stage in soybeans by linkage mapping approaches [12,13] For example, Zhang et al. [14] detected nine significant QTLs for flooding tolerance on chromosomes 1, 4, 5, 16, and 18 for both flooding tolerance scores (FTSs) and survival rates (SRs) using a ‘Benning × PI 416937’  recombinant inbred line population. Over 20 QTLs were also found in another RIL population ‘Danbaekkong × NTS1116’ [15,16]. These QTLs are distributed across 20 chromosomes in the soybean genome, indicating the complex QTL architecture for the soybean tolerance. However, some major loci such as *qWT_Gm03* on chromosome 3 for waterlogging tolerance and *Qrld-12* for root length development (RLD) or *Qrsad-12* for root surface area development (RSAD) on chromosome 12 have been confirmed to promote the stress tolerance [17,18].

In contrast to waterlogging tolerance research of soybean plants with a focus on plant injury and yield loss, the effect of seed-flooding tolerance at germination stage has not been well determined. Due to the excessive rainfall at sowing time in some areas, soybean cultivars with seed-flooding tolerance at germination stage are desirable for breeding. A previous study found no significant differences in the evaluation of seed-flooding tolerance between laboratory and soil experiment [19]. It is noteworthy that four integrated QTLs named *Sft1*, *Sft2*, *Sft3*, and *Sft4* were identified by evaluating germination rate (GR) and normal seedling rate (NS) at germination stage. *Sft2* on Chromosome 08 exhibited large effects on seed-flooding tolerance. Interestingly, this QTL was near *I* genetic locus which controls the seed coat pigmentation [20]. A total of 33 QTLs including 26 main-effect ones for the seed-flooding tolerance parameter of relative seedling length were identified in two related RIL populations with a common tolerance parent M8206 by using a restricted two-stage multi-locus multi-allele genome-wide association study [21]. By using a genome-wide association analysis (GWAS) method, 14 SNPs were found to be associated with the tolerance to waterlogging [22]. A total of 25 QTNs associated with GR, NS, and electric conductivity (EC) were detected in a germplasm population [23]. As one of the important indicators, the value of EC marked the intracellular substance leakage of soybean seeds under stress conditions, including flooding stress [24]. EC was also found to be closely related to the seed vigor, which reflects the seed germination and unhealthy seedling developments [25], and the germination test can be replaced by EC measurements carried out under soaking treatment [26,27]. Moreover, a total of 40 and 20 SNPs associated with GR and EC were found on a panel of 243 Plant Introduction from the USDA collection [28]. However, these QTLs are detected from a limited cultivated soybean background. It is essential to mine and detect stable quantitative trait loci (QTLs) and related candidate genes in more genetic backgrounds.

Compared with cultivated soybean (*Glycine max* (L.) Merr.), the annual wild soybean (*Glycine soja* Sieb. and Zucc.) displayed high genetic diversity and is acknowledged as the progenitor of cultivated soybean [29,30]. Previous studies demonstrated that the wild soybean not only has abundant oil [31] and protein content [32] but also exhibits strong adaptability and resistance/tolerance to various biotic and abiotic stresses [33,34,35,36], indicating that wild soybean can be used as the important source of genetic variability. Thus, we can improve the seed-flooding tolerance of cultivated soybean by introducing favorable alleles from wild soybean into elite cultivars. Wild soybean accessions were shown to be more waterlog-tolerant than cultivated soybean in a recent study [37]. Furthermore, the wild soybean accession PI342618B has demonstrated high seed-flooding tolerance [38], with the majority of seedlings surviving after a 140 h flooding treatment, and five candidate genes for the tolerance were screened out from differentially expressed genes of root tissue transcriptomic profile between PI342618B and a sensitive line [38]. By keeping the above in view, the objective of the present study was to identify QTLs for seed-flooding tolerance using two RIL populations derived by crossing common wild soybean accession PI342618B with two sensitive cultivated soybeans, NN86-4 and NN493-1, and then to mine candidate genes and elite alleles from *Glycine soja* in developing high seed-flooding tolerant soybean varieties.

## 2. Materials and Methods

### 2.1. Plant Materials

Two inter-specific RIL populations, NJIRNP and NJIR4P, consisting of 284 and 161 lines, respectively, were used for elucidating the genetic basis of seed-flooding tolerance. The NJIRNP and NJIR4P populations were derived by crossing the common seed-flooding tolerant wild soybean genotype PI342618B (*G. soja*) with two sensitive cultivated soybean varieties, NN86-4 (*G. max*) and NN493-1 (*G. max*). Parental accessions and two RIL populations were planted at the Jiangpu Experiment Station of Nanjing Agricultural University (32.12° N, 118.37° E), Jiangsu Province, China, in two consecutive growing seasons in 2013 and 2014, designated as JP13 and JP14. Both RIL populations were grown in a completely randomized block design with three replications.

### 2.2. Phenotypic Evaluation of Seed-Flooding Tolerance

For determining the optimum duration of seed-flooding treatment, 50 seeds were dipped into distilled water for different time points of 0, 2, 3, 4, 5, 6, and 7 days. Seeds of good quality were sterilized with 70% ethanol for 10 s to remove the contaminants. Then, seeds were rinsed with distilled water three times. After the water had evaporated naturally and the seeds became dry, flooding treatment was applied using 350 mL plastic cups with 100 mL distilled water at 25 °C and sterilized. Petri dishes were placed on top to cover the cups to prevent the water evaporation. The experiment was conducted in a completely randomized design with three replications. Based on the previous studies related to seed-flooding tolerance, both RIL populations and parental accessions were phenotypically evaluated for germination rate (GR) and normal seedling rate (NSR) [24]. Moreover, the electrical conductivity (EC) represented the content of seed substance leakage [25], and it was closely related to seed germination and survival rate [26,27]. Immediately after the treatment, a conductivity meter (model: DDS-307A) was utilized to record the EC value of steep-water plastic cups. A germination experiment was carried out using paper roll method [39]. Then, all the seeds were grown for 5 days in normal conditions, and the number of germinated seeds and normal seedlings were recorded. Seeds with radicle length more than 1 cm were regarded as germinated. Seedlings with normal cotyledon, radicle, and root were regarded as the normal seedlings. For control, seeds without flooding treatment were grown in the same conditions. The GR and NSR were estimated using the following equation:

GR = (germination rate under flooding treatment)/(germination rate in control) × 100%

NSR = (normal seedling rate under flooding treatment)/(normal seedling rate in control) × 100%

### 2.3. Analysis of Phenotypic Data

The analysis of variance (ANOVA) and descriptive statistics, including mean, range, standard deviation (SD), skewness, kurtosis, and correlations among three traits, were calculated using the SAS PROC generalized linear model (GLM), PROC UNIVARIATE, and PROC CORR programs, respectively [40]. The broad-sense heritability (*h*^2^) of RIL populations was estimated using the following equation:

$$h^{2}=\sigma_{G}^{2}/(\sigma_{G}^{2}+\sigma_{GE}^{2}/n+\sigma_{e}^{2}/nr)$$

where $\sigma_{G}^{2}$ is the genotypic variance, $\sigma_{GE}^{2}$ is the variance of the genotype-by-environment interaction, $\sigma_{e}^{2}$ is the error variance, n is the number of environments, and r is the number of replications within an environment [41].

### 2.4. QTL Mapping Analysis

Two high-density genetic linkage maps of NJRINP and NJRI4P populations contain 5728 and 4354 bin markers, respectively, constructed based on 89,680 and 80,995 single nucleotide polymorphisms (SNPs), and these SNPs were previously reported by Wang et al., 2016. Moreover, the total genetic distance covered was 2204.6 and 2136.7 cM for NJRINP and NJRI4P populations, with an average distance of 0.4 and 0.5 cM between neighboring bins, respectively [42]. The information of genetic and physical positions of all bin markers was presented (Supplementary Table S1).

The QTL analysis was performed via WinQTLCart 2.5 software [43] and QTLNetwork 2.2 [44]. For the WinQTLCart 2.5 software, the model of composite interval mapping (CIM) was used with a 10 cM window at a walking speed of 1 cM. The LOD threshold was calculated using 1000 permutations for an experimental-wise error rate of *p* = 0.05 to determine whether the QTL was significantly associated with traits [45]. For QTLNetwork 2.2, the model of mixed linear composite interval mapping (MCIM) was utilized to identify major additive effects QTLs, epistatic QTLs (AA), and genotype-by environment interaction effects (additive by environment (AE) and AA by environment (AAE)) [46].

### 2.5. qRT-PCR and Sequence Analysis

Soybean genomic data(Williams82 version) was downloaded from the Phytozome (http://phytozome.jgi.doe.gov) (accessed on 12 April 2014), SoyBase (http://www.soybase.org) (accessed on 13 April 2014), and SoyKB (http://soykb.org/) (accessed on 13 April 2014) websites. According to the physical regions related to seed-flooding, candidate genes were extracted from the predicted gene list based on the gene annotations (http://www.soybase.org) (accessed on 7 May 2014). The CDS sequencing was performed to verify the nucleotide mutation of candidate genes in three parental accessions, PI342618B, NN86-4, and NN493-1. The root parts of seedlings were collected for RNA extraction and qRT-PCR analysis. Total RNA was extracted by using the RNA Simple Total RNA kit (TIANGEN, Beijing, China) by following the manufacturer’s protocol. The genes were amplified by using Phanta^®^ Max Super Fidelity DNA Polymerase from Vazyme and sent to Generalbiol for sequencing. Amino acid homology alignment was analyzed using DNAMAN and BioXM 2.6. cDNA was synthesized using the Prime ScriptTM RT Reagent Kit (TaKaRa, Shiga, Japan) according to the manufacturer’s instructions. qRT-PCR(SYBR Green, FP207) was conducted to validate the differential expression levels of the candidate genes among three parental accessions. The experiment was run with three biological replicates, and an actin3-expressing gene was used as the internal control. All the primers were designed by Vector NTI 11.5 (Supplementary Table S2).

### 2.6. Vector Construction and Subcellular Localization

The method of homologous recombination was used for the vector construction in this study. The primers were designed based on the nucleotide sequence of *GmDREB2* (*Glyma.08g137600*) and the *XhoI* restriction site in *pJRH0641-GFP* (Supplementary Table S2). The full-length coding region of *GmDREB2* was introduced to *pJRH0641-GFP* for overexpression of *GmDREB2* driven by the *CaMV 35S* promoter (35S::*GmDREB2-GFP*), and the empty vector containing 35S:*GFP* was used as a control. The construction of the recombinant plasmid was conducted using In-Fusion^®^ HD Cloning Plus (TaKaRa, Japan) by following the manufacturer’s protocol. Then, the recombinant plasmid was transformed into *E.coli* DH5α and finally transformed into the *Agrobacterium tumefaciens* strain EHA105 by the electroporation method. The transient expression was carried out via the infection of *Agrobacterium* liquids containing the recombinant plasmid of *pJRH0641-GmDREB2::GFP* in young *N. benthamiana* leaves (5 weeks-old). After 36 h of the infection, the fluorescence in *N. benthamiana* leaves was detected using a confocal laser scanning microscope (Zeiss LSM780, Oberkochen, Germany).

### 2.7. Transformation of Soybean Hairy Roots

The 35S::*GmDREB2-GFP* overexpression vector was transformed into the *Agrobacterium rhizogenes* strain K599 for hairy root transformation by the electroporation method [42]. The cotyledonary node of young seedlings (one-week-old) with unfolded cotyledons were infected with *Agrobacterium rhizogenes* carrying the 35S::*GmDREB2-GFP*. The seedlings were transferred to the germination chamber with 16 h light/8 h dark cycle, at 25 °C, and with high humidity (>90%). When the emerged hairy roots grew up to 5–10 cm after approximately 3 weeks, the primary roots were removed. The transgenic positive hairy roots were screened by detecting the GFP signals using the fluorescence microscope. Then, the transgenic positive hairy roots were grown under hydroponics cultivation using 1/2 Hoagland nutrient solution for five days and finally treated with flooding stress for 4d. The fresh weight of hairy roots was tested by the analytical balance.

## 3. Results

### 3.1. Determination of Optimum Seed-Flooding Treatment Time Point

**Figure 1:**
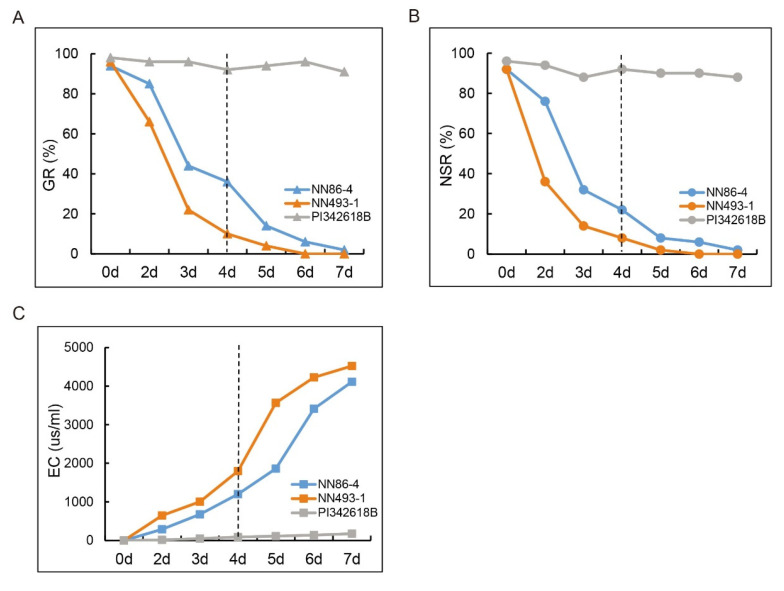
Germination rate (GR), normal seedling rate (NSR) and electrical conductivity (EC) of three parental accessions (NN86-4, NN493-1, and PI342618B) at various time points (0, 2, 3, 4, 5, 6, and 7 d) of flooding treatment. (**A**) GR. (**B**) NSR. (**C**) EC. The dotted lines indicate the optimum treatment time point.

**Figure 2:**
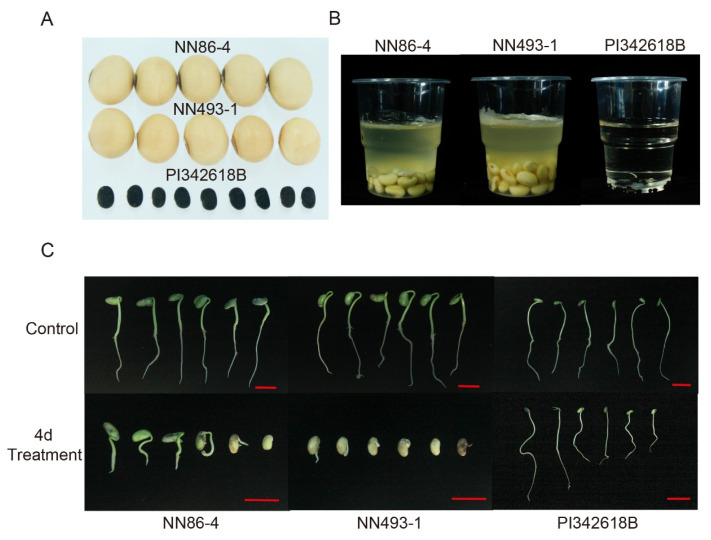
Phenotype and germination performance of three parental accessions NN86-4, NN493-1, and PI342618B. (**A**) Seed size of three parents. (**B**) The morphology of seeds in plastic cups after 4 d flooding treatment. (**C**) The germination and growth performance of three parents that were initially subjected to 4 d flooding treatment under normal growth conditions for 5 days. Scale bar indicates 2.0 cm.

Optimum seed-flooding treatment was determined by the evaluation of germination rate (GR), normal seedling rate (NSR), and electrical conductivity (EC) for three parents, viz., NN86-4, NN493-1, and PI342618B at different treatment time points (0, 2, 3, 4, 5, 6, and 7 days). Both GR and NSR of NN86-4 and NN493-1 were markedly decreased after 3 days of treatment, while most seeds of PI342618B germinated very well (Figure 1A,B), The GR and NSR of NN86-4 and NN493-1 were continuously reduced with the increasing treatment time after 4 days of treatment, and they were both maintained at a low level (<15%) after 5 days of treatment. In contrast, the EC of NN86-4 and NN493-1 rapidly increased along with the treatment time, suggesting more leakage of electrolytes from the seeds, while the EC of PI342618B was constantly maintained at a lower level (Figure 1C). Furthermore, the phenotype and germination performance of three parents are shown in Figure 2. The seedling growth of PI342618B was considerably better than that of NN86-4 and NN493-1 under 4 d flooding treatment (Figure 2C), indicating that it was advantageous to differentiate the performance of these three parents under 4d treatment. In conclusion, the above three germination-related parameters revealed a significant difference between seed-flooding tolerant (PI342618B) and sensitive (NN86-4 and NN493-1) soybean parents under 4 d flooding treatment (Figure 1 and Figure 2). Hence, we selected 4 days as the optimum flooding treatment time for the evaluation of seed-flooding tolerance in the present study.

### 3.2. Evaluation of Phenotypic Variation

The seed size of NN86-4 and NN493-1 was much bigger than that of PI342618B. The external appearance of parental seeds after 4 d of flooding treatment in the plastic cups is presented in Figure 2B. The phenotypic data analysis of three parents is shown in Table 1. The GR and NSR of PI342618B were significantly higher relative to those of NN86-4 and NN493-1 in both JP13 and JP14 environments, whereas the EC of PI342618B was considerably lower. These results indicated that PI342618B was more tolerant to seed-flooding stress compared to cultivated parents NN86-4 and NN493-1, and NN86-4 was relatively more tolerant than NN493-1. Additionally, the phenotypic performance of NJRINP and NJRI4P populations was observed (Table 2). Descriptive statistics, ANOVA (*F*-value), and estimates of heritability were performed for all three traits related to seed-flooding tolerance in both populations under JP13 and JP14 environments (Table 2). The heritability estimate (*h*^2^) of GR, NSR, and EC across both years ranged from 0.67 to 0.89. High positive significant correlations (*r* = 0.89 to 0.96, *p* < 0.001) were observed between GR and NSR across environments in both populations, while both GR and NSR were negatively correlated with EC (*r* = −0.31 to −0.81, *p* < 0.001) (Table 3). The ANOVA results displayed that the difference between RILs for both populations was highly significant for all three traits (*p* < 0.01) (Supplementary Table S3). The environmental differences and genotype-by-environment (G × E) interaction effects were also significant for these three traits (*p* < 0.01).

### 3.3. QTL Mapping of Seed-Flooding Tolerance by CIM

The QTL identification of GR, NSR, and EC was performed using high-density genetic maps of NJIRNP and NJIR4P populations. As shown in Table 4, 25 QTLs explaining 3.53–30.58% of the phenotypic variation (*R*^2^) associated with seed-flooding tolerance were detected via CIM in different populations and environments. Eight QTLs associated with GR were identified on five chromosomes. Among these QTLs, two were located on Chromosome 6; three were located on Chromosome 8, and the remaining were located on Chromosome 1, Chromosome 17, and Chromosome 20, respectively (Table 4). These eight QTLs explained 3.71 to 30.58% of phenotypic variance (PV). Notably, the most prominent QTL with the highest LOD score (25.14) was identified on Chromosome 8, which is contained in *qGR-8-2*, explaining 30.58% of PV. Additionally, *qGR-8-2* was further detected in both populations and environments. For NSR, seven QTLs were identified on three chromosomes. Out of these QTLs, two were located on Chromosome 6, three were located on Chromosome 8, and the remaining two were located on Chromosome 15. The QTL with the highest LOD score (23.56) was identified in the *qNSR-8-2* region, explaining 28.93% of PV. Moreover, ten QTLs for EC were identified on seven chromosomes. Out of these QTLs, *qEC-8-1*, *qEC-8-2*, and *qEC-8-3* detected on Chromosome 8 were located in the same mapping region as detected for GR and NSR. In addition, *qEC-8-2* was identified in both populations as well as in different environments, explaining 6.63–21.12% of PV, and one QTL in the *qEC-8-2* region displayed the largest effect with *R*^2^ = 21.12%. The remaining seven QTLs were only detected in a single population or environment (Table 4).

All the QTLs identified for GR and NSR in both populations displayed negative effects, and all of the positive alleles were from the tolerant parent PI342618B. Similarly, all the QTLs for EC revealed positive effects, suggesting the positive alleles that reduced the electrolyte leakage from seeds came from tolerant wild parent. By comparing the physical regions of QTLs for three traits detected on Chromosome 8, we found that all these QTLs were extremely close and located in three genomic regions, indicating that there were QTL clusters/hotspots related to seed-flooding tolerance on Chromosome 8. Moreover, most of these QTLs within these clusters displayed large effects with *R*^2^ > 10%, suggesting that Chromosome 8 was most likely rich in genes involved in seed-flooding tolerance in soybean.

### 3.4. QTL Mapping by MCIM and Comparative Analysis of CIM and MCIM Methods

To further validate the QTLs detected by CIM, we performed MCIM to dissect the additive QTLs. A total of 18 major additive QTLs for GR, NSR, and EC were identified in both populations, and these QTLs were distributed on seven chromosomes (Table 5). These additive QTLs explained 2.0–30.15% of the PV. For GR, five QTLs were identified, including *qGR-8-1* and *qGR-8-3* detected in same genomic regions on Chromosome 8 as reported through CIM. The *qGR-8-1* was identified in both populations, accounting for 20.42% and 20.60% of the PV, respectively. For NSR, four additive QTLs were detected and one of these QTLs; *qNSR-8-1* exhibited large additive effects and explained 30.15% and 24.00% of the PV in NJIRNP and NJIR4P, respectively. For EC, nine QTLs were identified, and all of these QTLs were only detected in a single population either NJIRNP or NJIR4P. Among them, *qGR-8-1* and *qGR-8-2* were located in the same genomic regions on Chromosome 8 as reported through CIM. Interestingly, we observed that stable QTLs (*qGR-6-1*, *qGR-6-2*, *qNSR-6-1*, *qNSR-6-2*, *qEC-6-1* and *qEC-6-2*) were mapped in the same regions on Chromosome 6 only in the NJIR4P population, and most of these QTLs can be verified through CIM. Additionally, all the positive alleles of QTLs for GR and NSR were derived from wild parent PI342616B, while the additive effects of QTLs for EC were negative, indicating that the positive alleles of QTLs for EC were from cultivated parents NN86-4 and NN493-1.

Furthermore, the epistasis and QTL-by-environment (Q×E) interactions were performed in this study. A total of four pairs of digenic epistatic QTLs were identified for NSR and EC (Table 6). In the NJIRNP population, only one pair of epistatic QTLs for EC was identified, and it consisted of two non-additive effect QTLs (*qEC-9-1* and *qEC-20-1*), explaining 3.42% of the PV. Moreover, in the NJIR4P population, three pairs of epistatic QTLs were detected. Out of these, one pair was identified for NSR which consisted of two non-additive QTLs (*qNSR-3-1* and *qNSR-9-1*), accounting for 5.03% of the PV. The remaining two pairs were identified for EC, and one pair consisted of two additive QTLs, viz., *qEC-4-1* and *qEC-8-1*, explaining 3.20% of the PV. Another pair was composed of two additive *qEC-3-1* and *qEC-15-1* and explained 2.54% of the PV.

In conclusion, we performed a comparative analysis of major QTLs associated with seed-flooding tolerance through CIM and MCIM. A total of 25 and 18 QTLs were identified by these two methods. Among these QTLs, 12 QTLs were commonly detected by both methods, indicating that these QTLs were stable and reliable. It is noteworthy that an important genomic region for seed-flooding tolerance was identified on Chromosome 8, which contains major QTLs for all three traits GR, NSR, and EC detected in both populations and environments by two methods. Furthermore, we also found stable genomic regions harboring QTLs for all three of the above traits on Chromosome 6, and these QTLs could be validated by both CIM and MCIM. However, these QTLs on Chromosome 6 were only identified in NJIR4P population, and most of these QTLs exhibited minor effects (*R*^2^ < 10%). In summary, we obtained a stable major genomic region associated with all three traits (GR, NSR, and EC) on Chromosome 8, and this region was verified in various populations and environments, as well as different mapping methods. Hence, this critical genomic region on Chromosome 8 can be considered as a potential candidate genomic region for breeding high seed-flooding tolerant soybean.

### 3.5. QTL Hotspots for Seed-Flooding Tolerance

QTL clusters/hotspots are defined as densely populated QTL regions on the chromosome that contain multiple QTLs associated with various traits. Based on the genetic position of major QTLs of GR, NSR, and EC, we observed that QTLs related to these traits were consistently located in the same or similar genomic regions. By comparing the mapping results using CIM, QTL hotspots on Chromosome 6 and Chromosome 8 were identified (Table 7). We found three QTL “hotspots 8-1, 8-2 and 8-3” on Chromosome 8, and these regions were adjacent to each other but non-overlapping (Table 7). The QTL “hotspot 8-1” harbors three QTLs related to all three traits and covers the physical length of 1.13 Mb. Likewise, QTL “hotspot 8-2” contains three QTLs within the physical interval of 1.35 Mb on Chromosome 8. Moreover, QTL “hotspot 8-3” possessed two QTLs within a physical genomic interval of 0.75 Mb. Among these three QTL hotspots on Chromosome 8, QTL “hotspot 8-2” containing multiple QTLs associated with all three traits has been detected in all studied environments and populations as well as mapping methods (Table 4 and Table 5), while “hotspots 8-1 and 8-3” were only identified in a single population. Furthermore, all the QTLs underlying QTL “hotspot 8-2” were major QTLs with *R*^2^ > 10% (Table 7). In addition, two QTL “hotspots 6-1 and 6-2” on Chromosome 6 containing two/three QTLs related to two/three studied traits were only detected in the NJIR4P population under a single environment, and all the QTLs underlying these two QTL hotspots displayed minor effects (*R*^2^ < 10%) (Table 7). In conclusion, the major QTL “hotspot 8-2” located on Chromosome 8 was considered as the major genomic region governing the inheritance of seed-flooding tolerance in soybean and can be utilized as the major targets for fine mapping, candidate gene identification, and marker-assisted breeding.

### 3.6. Genetic Association between Seed-Flooding Tolerance and Seedcoat Pigmentation

An earlier study reported that seed-coat color was closely linked with seed-flooding tolerance, and varieties with a pigmented seed-coat displayed higher tolerance than those with yellow seed-coat [19]. In this study, we observed that a large proportion of NJIRNP and NJIR4P populations showed various pigmented seed-coat colors, including black, brown, green, and other mixed colors. To identify the potential association between seed-flooding tolerance and seed-coat pigmentation, the effects of seed-coat color on seed-flooding tolerance were investigated. The results showed that the GR and NSR of the genotypes with pigmented seed-coat in both NJIRNP and NJIR4P were significantly higher than those of genotypes with yellow seed-coat (Table 8). Additionally, the GR and NSR of the genotypes with black color were considerably higher compared to the genotypes with other pigmented colors. Moreover, the EC of pigmented varieties was remarkably lower than that of yellow varieties, suggesting that less seed electrolyte leakage occurs in pigmented soybean genotypes under flooding treatment. In conclusion, all of this evidence supports the view that varieties with pigmented seed-coat tended to be more seed-flooding tolerant and genotypes with black seed-coat color displayed the largest tolerance.

In soybean, the *I* locus plays a critical role in the inhibition of seed-coat pigmentation, and it was identified as a genomic region of unusual cluster arrangement of three chalcone synthase genes, viz., *CHS1*, *CHS3*, and *CHS4* on Chromosome 8 [47]. 

Interestingly, the QTL hotspots on Chromosome 8 identified in the present study associated with seed-flooding tolerance were also detected near the *I* locus. Based on the close association between seed-flooding tolerance and seed-coat pigmentation described above, we hypothesized that these three *CHS* genes might be the candidate genes for seed-flooding tolerance. To further clarify the sequence differences of *CHS1*, *CHS3*, and *CHS4*, we sequenced these genes in PI342616B, NN86-4, and NN493-1. Subsequently, two, six, and one nucleotide variations were identified in *CHS1*, *CHS3*, and *CHS4*, respectively (Supplementary Table S4). However, all these nucleotide variations were synonymous mutations and did not result in any changes to the amino acid sequence of the corresponding proteins (Supplementary Figure S1). By combining the above results, it was revealed that the QTL hotspots and *I* locus were located in the same genomic region on Chromosome 8, but *CHS1*, *CHS3*, and *CHS4* were not the candidate genes involved in seed-flooding tolerance.

### 3.7. qRT-PCR and Sequence Analysis of Candidate Genes

**Figure 3:**
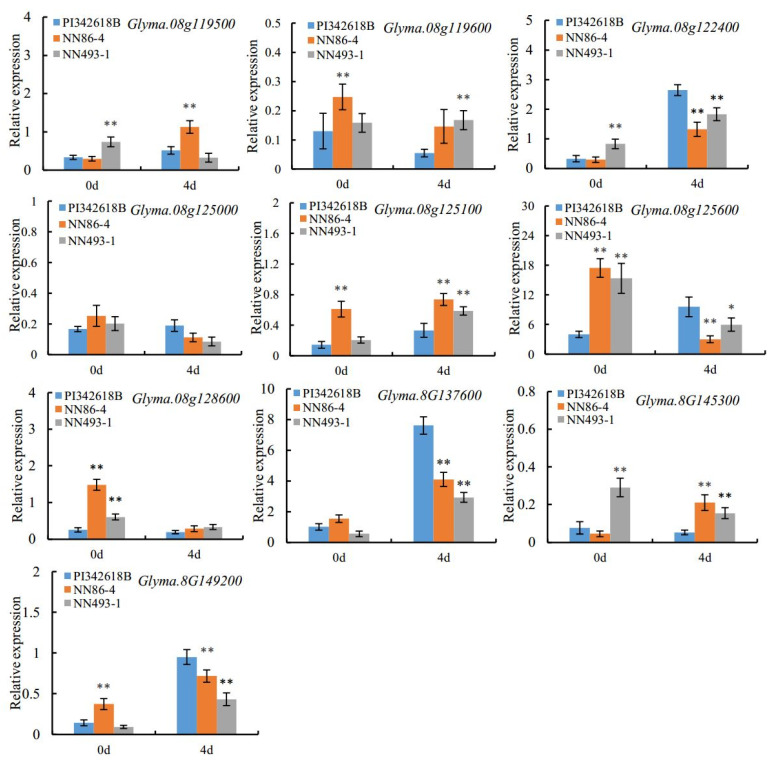
Relative expression level of ten candidate genes in PI342618B, NN86-4, and NN493-1 under 4 d flooding treatment. Asterisks on the top of bars indicate statistically significant differences (*p* < 0.01).

As the major QTL “hotspot 8-2” associated with all traits was consistently identified by both methods as well as in multiple environments and populations, the candidate gene prediction analysis was further performed in this region. All the model genes and their annotations were downloaded from Phytozome (https://phytozome.jgi.doe.gov) (accessed on 17 May 2014), Soybase (http://www.soybase.org) (accessed on 17 May 2014), and SoyKB (http://soykb.org/) (accessed on 18 May 2014), databases, and a total of 164 genes were identified within QTL “hotspot 8-2”. In order to identify key candidate genes controlling seed-flooding tolerance, the expression levels of these 164 genes in different soybean tissues were examined using the RNA-seq data from Soybase (https://www.soybase.org/soyseq/) (accessed on 24 May 2014), (Figure S2). As the root was an important plant organ in response to flooding stress [48], candidate genes with high expression in roots were selected. Additionally, previous study revealed that flooding tolerance was potentially associated with carbohydrate catabolism and cell wall loosening [49]. Moreover, genes encoding glycosyl transferase proteins, cytochrome P450, and ERF/AP2 transcription factors were reported to be involved in flooding stress during germination [50]. By combining the analysis of gene expression, functional annotation, and the published literature, a total of ten candidate genes were screened (Supplementary Table S5). To confirm the expression of these genes under flooding treatment, we performed qRT-PCR analysis. The results revealed that five of these predicted candidate genes, *Glyma.08G122400*, *Glyma.08g125100*, *Glyma.08G137600*, *Glyma.08g145300*, and *Glyma.08G149200* were significantly induced under flooding treatment compared to the control in all three parental accessions (Figure 3), indicating that these genes may play key roles in response to flooding stress. Moreover, all these three up-regulated genes displayed relatively high expressions in seed-flooding tolerant parent PI342618B compared to sensitive parents (NN86-4 and NN493-1). Based on these findings, these three differentially expressed genes were initially considered as the possible candidate genes for seed-flooding tolerance.

**Figure 4:**
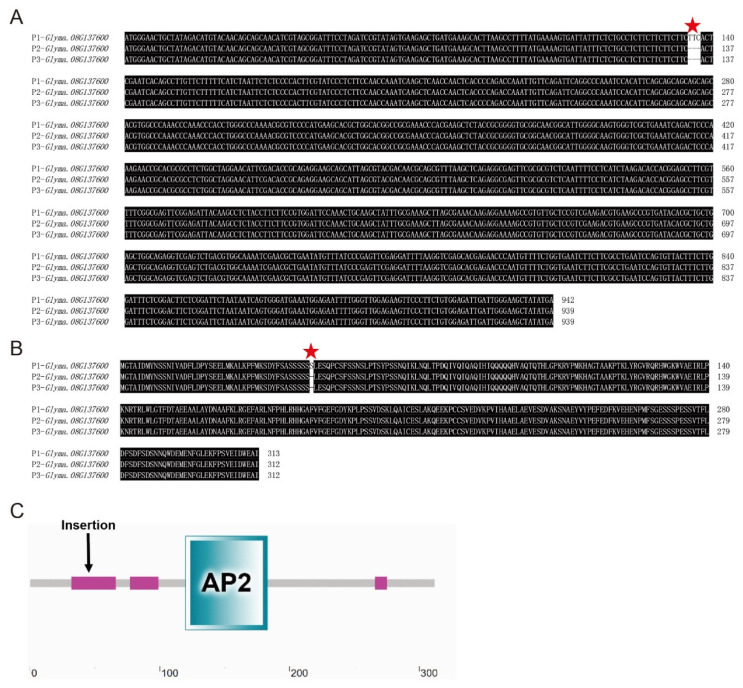
Nucleotide and amino acid sequence alignment of *Glyma.08G137600* in three parents and the domain composition of *Glyma.08G137600*. (**A**) The multiple nucleotide sequence alignment of *Glyma.08G137600* in P1(PI342618B), P2 (NN86-4), and P3 (NN493-1). (**B**) The multiple amino acid sequence alignment of *Glyma.08G137600* in P1, P2, and P3. (**C**) The insertion location in *Glyma.08G137600*. The red star represents the position of nucleotide or amino acid mutations.

To further verify the above hypothesis, we performed a sequence analysis of the full-length coding region of these three candidate genes in three parents, viz., PI342616B, NN86-4, and NN493-1. By comparing the sequence differences, a single base mutation (G/A) in the CDS region of *Glyma.08G122400* in wild parent PI342616B was observed (Supplementary Figure S3A), and this synonymous mutation did not result in any changes in amino acid (Supplementary Figure S3B). Additionally, *Glyma.08G137600* exhibited three base insertions (-/TTC) in PI342616B (Figure 4A). This mutation caused the insertion of serine in the low complexity region of protein (Figure 4B,C). However, no nucleotide changes were detected in *Glyma.08g125100*, *Glyma.08g145300*, and *Glyma.08G149200*. Based on the above results of qRT-PCR and sequence analysis, *Glyma.08G137600* was considered as the most possible candidate gene for seed-flooding tolerance, and this gene was designated as *GmDREB2* for further study.

### 3.8. Subcellular Localization and Transgenic Hairy Roots Verification of GmDREB2

**Figure 5:**
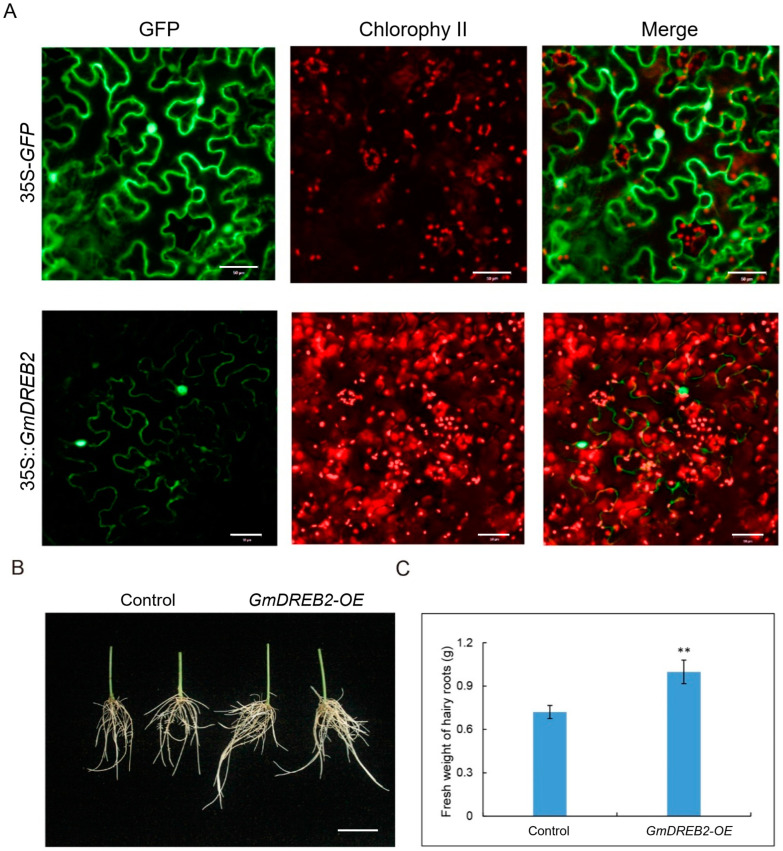
The subcellular localization of *GmDREB2* in the leaf of *N. benthamiana* and performance of *GmDREB2* transgenic hairy roots. (**A**) The subcellular localization of *GmDREB2*. Scale bar indicates 50 μm. (**B**) Growth of *GmDREB2* transgenic hairy roots under 4 d flooding treatment. Scale bar indicates 5 cm. (**C**) Fresh weight of *GmDREB2-OE* and control hairy roots. ** indicates statistically significant differences (*p* < 0.01).

To reveal the subcellular localization of the *GmDREB2* protein, we performed the transient expression of 35S::*GmDREB2*-GFP in fusion with GFP in *N.benthamiana* leaves. Based on the prediction of subcellular localization on SoftBerry (http://linux1.softberry.com/), *GmDREB2* was localized in the nucleus and plasma membrane. As shown in Figure 5A, the green fluorescence of *GmDREB2*-GFP was mainly distributed in the nucleus and plasma membrane, which confirmed that *GmDREB2* protein was localized to the nucleus and plasma membrane.

Additionally, to investigate the role of *GmDREB2* under flooding stress, 35S::*GmDREB2*-GFP was generated for overexpression (*GmDREB2*-OE) analyses in the hairy roots. The transgenic soybean hairy roots were generated via the *Agrobacterium rhizogenes*-mediated hairy root transformation system [51], and the susceptible cultivated parent NN493-1 was used as the transgenic receptor material. In order to avoid the wilting of hairy roots, all the transgenic hairy roots were immersed in the liquid, and no difference in the hairy roots was observed between *GmDREB2*-OE and the control before treatment. As shown in Figure 5B, a significant difference was found, and the hairy roots of *GmDREB2*-OE displayed relatively better growth than the control after 4 d of flooding stress. Moreover, the fresh weight of the *GmDREB2*-OE hairy roots was significantly heavier than that of the control (Figure 5C). These preliminary results reveal that the overexpression of *GmDREB2* can promote the growth of soybean hairy roots under flooding stress. However, further validation is still required to verify the molecular function of *GmDREB2* in seed-flooding tolerance in soybeans.

## 4. Discussion

Soybean was found to be relatively sensitive to flooding stress during germination, vegetative, and reproductive stages in a previous study [52]. It is an effective way to reduce the impact of flooding stress on soybean production by screening elite flooding tolerant germplasm and breeding high tolerant varieties [53]. However, the flooding tolerance of soybeans is a complex quantitative trait governed by multiple loci/genes with high environmental influence [14,20]. Over the past decades, 27 QTLs have been detected and reported on SoyBase using bi-parental mapping populations, which are distributed across the 20 chromosomes in the soybean genome, with several more recently detected [14,16]. Yu et al. (2019) identified 25 and 21 quantitative trait nucleotides (QTNs) by the mixed linear model (MLM) and multi-locus random-SNP-effect mixed linear model (mrMLM) of GWAS [23], respectively. The two separate models for germination rate (GR), electrical conductivity (EC), and normal seedling rate (NSR) detected QTN13 on chromosome 13. *Glyma.13g248000* (*GmSFT*), a QTN13-predicted candidate gene, was discovered to have a nonsynonymous mutation in seed-flooding tolerant genotypes, leading to an amino acid substitution in the protein. However, some major QTLs could be effectively utilized in breeding improved flooding-tolerant varieties or identifying candidate genes using marker-assisted selection (MAS). In this context, to better dissect the genetic basis of seed-flooding tolerance in soybean, two inter-specific high-density linkage maps of NJRINP and NJRI4P populations were utilized to identify major and stable QTLs related to seed-flooding tolerance using three parameters (GR, NSR, and EC) at the germination stage. Furthermore, two different methods, CIM and MCIM, were performed to improve the accuracy of mapping results. A total of 25 and 18 QTLs were detected by CIM and MCIM, respectively. Among these QTLs, 12 common QTLs were verified through both CIM and MCIM, indicating that these QTLs were stable and could be effectively utilized as potential candidate regions for enhancing seed-flooding tolerance. Interestingly, the mapping results displayed that QTLs related to various traits were distributed as clusters, and QTL hotspots of seed-flooding tolerance were identified on Chromosome 6 and Chromosome 8. We detected two QTL hotspots “6-1 and 6-2” on Chromosome 6. All the QTLs contained in hotspot “6-1”and hotspot “6-2” were only identified in a single population (NJIR4P) and explained small phenotypic variation with *R*^2^ < 10.0%, indicating that these loci were minor QTLs related to seed-flooding tolerance.

Among the identified QTL hotspots, QTL hotspot “8-2” possessing QTLs associated with all three traits was detected in both populations and environments, while QTLs within hotspot “8-1”and hotspot “8-3” were only detectable in single populations (NJIR4P or NJIRNP). Furthermore, QTL hotspot “8-2” was validated by both CIM and MCIM, and all the QTLs contained in “hotspot 8-2” displayed large effects on seed-flooding tolerance (*R*^2^ > 10.0%). Some previous studies also displayed major QTLs related to seed-flooding tolerance in this map region [20,28]. For example, Sayama et al. (2009) identified *sft2* near the *I* locus on chromosome 8 (LG_A2) [20], which is involved in seed coat pigmentation. Therefore, it was concluded that QTL hotspot “8-2” was the most likely candidate region for seed-flooding tolerance. Besides the QTL hotspots detected on Chromosome 6 and Chromosome 8, we also identified some QTLs on other chromosomes, including Chromosome 1, Chromosome 3, Chromosome 15, and so on. By comparing the genomic position of these QTLs with the previously identified loci, *qGR-1-1* and *qEC-1-1* are overlapped with the *Flood tolerance 6-1* locus coded on the SoyBase website detected by Rizal and Karki [54], and the region of *qEC-17-1* on Chromosome 17 also includes *QTL SFT_17-52* and *RSL-a-17-1a* found by Dhungana et al. [16] and Ali et al. [21].

The genetic makeup of complex traits is not only regulated by main-effect QTLs/genes but also by inter-locus interactions as well as their interactions with the environment [55]. Understanding the epistasis and QTL × environment (QE) interaction effects are essential for revealing the crucial genetic basis for determining quantitative traits and improving QTL detection and selection accuracy. Hence, ignoring inter-genic interaction will result in an overestimation of individual QTL effects and an underestimation of genetic variance [56]. In this study, four pairs of digenic epistatic QTLs were detected, three for EC and one for NSR. Out of these, only one epistatic QTL pair (*qEC-4-1* and *qEC-8-1*) displayed individual additive effects, while the remaining three epistatic QTL pairs showed non-additive effects. These three epistatic QTL pairs might serve as modifying genes that themselves have no significant effects but regulate the expression of flooding-tolerance related genes through epistatic interactions. All four pairs have significant AA effects. However, the total PVE explained by four epistatic pairs related to seed-flooding tolerance was about 14.19%. Similar results for the epistatic interaction of QTLs for different traits in soybean have been reported in early studies [57,58]. The presence of epistatic interactions for a given trait makes selection more difficult. Interestingly, all main-effect QTLs identified in the present study, except for *qEC-4-1* and *qEC-8-1*, had no epistatic interactions, which increased the heritability of the trait and made selection easier.

A previous study found a strong correlation between seed-flooding tolerance and seed-coat color in soybean [19]. Moreover, early study also demonstrated that the seed-flooding of pigmented genotypes was significantly more tolerant compared to genotypes with yellow seed-coat [20]. Therefore, these results potentially indicated that *Sft2* and *I* were the same locus or that they possessed strong linkage with each other [20]. Similar results were observed in the present study that genotypes with pigmented seed-coat (black, brown, green, and other pigmented colors) exhibited more seed-flooding tolerance than those with yellow seed-coat in NJIRNP and NJIR4P populations. These findings provide further evidence that the seed-coat pigmentation is strongly associated with seed-flooding tolerance. Interestingly, three identified QTL “hotspots 8-1, 8-2 and 8-3” for seed-flooding tolerance in our study were located near the *I* locus on Chromosome 8. It is known that the *I* locus controlling seed-coat pigmentation encompasses a region of three duplicated chalcone synthase genes (*CHS1*, *CHS3*, and *CHS4*) in soybean [59]. Thus, to exclude the possibility that these three *CHS* genes were the candidate genes involved in seed-flooding tolerance, we performed the sequence analysis of *CHS* genes between PI342616B and NN86-4, NN 493-1. The results revealed no changes were observed in the amino acid sequence of *CHS* proteins among parents, indicating that the *CHS* pathway may be independent of seed-flooding tolerance. Furthermore, our additional evidence supported this viewpoint because we observed many accessions with the yellow seed-coat exhibiting high GR and NSR in both populations in the present study.

So far, limited studies have been carried out to identify candidate genes for seed-flooding tolerance in soybean. In this regard, mining of the candidate genes for seed-flooding tolerance in soybean revealed 164 model genes within the physical intervals of QTL “hotspot 8-2” on Chromosome 8 in the present study. Based on the expression levels of these genes in different soybean tissues from Soybase (https://www.soybase.org/soyseq/) and the functional annotations related to seed-flooding tolerance, ten candidate genes were screened. The qRT-PCR and sequence analysis were further performed, and the results revealed that *Glyma.08G137600* was significantly induced under 4 d of flooding stress in all three parents (PI342618B, NN86-4, and NN493-1) compared with the control. Moreover, three consecutive base insertions (-/TTC) in the coding region of PI342616B were observed, and this mutation resulted in the insertion of serine in the low complexity region of protein. These results indicated that *Glyma.08G137600* was the most likely possible candidate gene for seed-flooding tolerance in soybean, and this gene was designated as *GmDREB2*. The functional annotation of *GmDREB2* exhibited that it encodes the ERF transcription factor, which contains the AP2 domain. In rice, an ethylene response factor (ERF) gene named *Sub1A* was characterized and involved in submergence tolerance [60]. Moreover, two ethylene response factors *SNORKEL1* and *SNORKEL2*, which play vital roles in response to deepwater stress by ethylene signaling, were identified [61]. In Arabidopsis, it was demonstrated that three ERF-ⅦII transcription factors (*RAP2.2*, *RAP2.3*, and *RAP2.12*) were involved in regulating and activating the core anaerobic response [62]. All the above functional studies illustrate the important role of the ERF transcription factor in flooding stress. Thus, *GmDREB2* was considered the most likely candidate gene for seed-flooding tolerance in soybean in the present study. The results demonstrated that the overexpression of *GmDREB2* relatively promoted the growth of soybean hairy roots under flooding stress, indicating that *GmDREB2* was positively involved in the regulation of seed-flooding tolerance in soybean. However, it requires more verification experiments to validate the molecular function of *GmDREB2* for its specific roles in seed-flooding tolerance.

In the present study, we reported that the wild parent PI342616B exhibited strong seed-flooding tolerance compared to the sensitive cultivar parents NN86-4 and NN493-1 under flooding stress. Moreover, all the favorable positive alleles of identified QTLs were derived from PI342616B, which supported the view that wild soybean gene pool is an abundant source of elite stress-tolerant genes. Wild soybean, as the progenitor of cultivated soybean, has been reported to display high contents of oil and protein, rich in genetic diversity, strong adaptability, and tolerance to various stresses [21,22,23]. Due to the favorable tolerance alleles contributed by wild soybean, it has become an efficient way to improve the seed-flooding tolerance of elite soybean varieties by introducing the seed-flooding tolerant QTLs/genes from wild soybean into elite cultivars.

## 5. Conclusions

The present study was a detailed investigation into uncovering the genetic basis of seed-flooding tolerance in soybean, in which we used high-density inter-specific genetic maps of two RIL populations (NJIRNP and NJIR4P) for identifying QTLs associated with seed-flooding tolerance. In total, we identified 25 and 18 QTLs by CIM and MCIM, respectively. Most of the identified QTLs were novel loci, and all the positive alleles were derived from wild parent PI342618B. Interestingly, one major QTL “hotspot 8-2” containing many QTLs related to all three traits was identified on Chromosome 8. Based on the gene expression and function annotation analysis of model genes within the QTL “hotspot 8-2”, ten candidate genes were selected. The results of qRT-PCR and sequence analysis demonstrated that *GmDREB2*(*Glyma.08G137600*) was the most possible candidate gene responsible for seed-flooding tolerance in soybean. *GmDREB2* encodes an ERF transcription factor and is localized in the nucleus and plasma membrane. Moreover, the overexpression of *GmDREB2* significantly promoted the growth of soybean hairy roots under flooding stress, indicating that *GmDREB2* plays an important role in the regulation of seed-flooding tolerance in soybean. However, it needs further validation to elucidate the functional roles of *GmDREB2* in seed-flooding tolerance. Finally, our findings will be useful for marker-assisted breeding of elite seed-flooding tolerant soybean varieties, as well as increasing our understanding of the genetic control of seed-flooding tolerance in soybean.

## Figures and Tables

**Table 1:** Phenotypic data of GR, NSR, and EC for three parents NN86-4, NN493-1, and PI342618B under 4 d flooding treatment in JP13 and JP14 environments.

Env.	Parent	GR	NSR	EC (us/mL)
JP13	NN86-4	0.37	0.25	1133.4
	NN493-1	0.11	0.08	1860.0
	PI342618B	0.93	0.89	107.9
JP14	NN86-4	0.33	0.21	1221.7
	NN493-1	0.06	0.05	1712.7
	PI342618B	0.88	0.86	99.0

NN86-4 and NN493-1 represent Nannong86-4 and Nannong493-1. GR, Germination Rate; NSR, Normal Seedling Rate; EC, Electrical Conductivity.

**Table 2:** Descriptive statistics, analysis of variance (ANOVA) and heritability of two RIL populations, NJRINP and NJRI4P, in JP13 and JP14 environments.

Population	Trait	Env.	Mean	SD	Range	Skewness	Kurtosis	*F*	*h* ^2^
NJRINP	GR	JP13	0.76	0.16	0–0.93	0.63	1.53	3.41 ***	0.67
		JP14	0.72	0.24	0–1.00	−0.47	−0.28	3.76 ***	
	NSR	JP13	0.68	0.15	0–0.87	0.31	3.04	6.79 ***	0.84
		JP14	0.66	0.20	0–0.81	1.35	8.62	13.35 ***	
	EC	JP13	189.7	77.5	15.0–1351.0	5.7	53.94	6.40 ***	0.89
		JP14	209.8	86.4	9.8–2208.0	2.19	5.52	14.18 ***	
NJRI4P	GR	JP13	0.63	0.19	0–0.98	−0.47	−1.37	6.75 ***	0.78
		JP14	0.71	0.22	0–0.92	−0.52	−0.82	4.83 ***	
	NSR	JP13	0.56	0.15	0–0.87	−0.07	−1.49	4.50 ***	0.8
		JP14	0.64	0.21	0–0.94	−0.23	−1.03	22.59 ***	
	EC	JP13	321.6	104.9	19.0–1957.5	1.59	2.97	20.84 ***	0.82
		JP14	266.8	80.2	14.5–1235	1.77	3.94	5.72 ***	

Env. represents environment. *** represents significant level at *p* < 0.001. *F* value represents ratio of mean squares. *h*^2^ represents broad sense heritability estimate.

**Table 3:** Phenotypic correlations (*r*) among three seedling-flooding tolerance related traits GR, NSR, and EC in NJRINP and NJRI4P populations in two environments.

NJRINP	GR	NSR	EC	NJRI4P	GR	NSR	EC
GR	1	0.94 ***	−0.32 ***	GR	1	0.96 ***	−0.79 ***
NSR	0.89 ***	1	−0.31 ***	NSR	0.97 ***	1	−0.75 ***
EC	−0.76 ***	−0.67 ***	1	EC	−0.81 ***	−0.80 ***	1

The datapoints under and above the diagonal represent r values of Jiangpu 2013 on the left and Jiangpu 2014 on the right, respectively. *** indicates significant level at *p* < 0.001.

**Table 4:** QTLs associated with GR, NSR, and EC detected by CIM in NJIRNP and NJIR4P populations in JP13 and JP14 environments.

Trait	QTL	Pop-Env. ^a^	Chromosome ^b^	QTL Peak Position (cM)	Peak Position Marker	LOD ^c^	1-LOD Interval (cM) ^d^	Physical Interval (bp)	*R*^2^ (%) ^e^	Additive Effect ^f^
GR	*qGR-1-1*	NP-JP14	1	3.83	BIN10	3.76	2.42–4.72	643,853–1,458,641	3.71	−0.07
	*qGR-6-1*	4P-JP14	6	30.94	bin62	5.03	28.96–32.14	6757,892–7,896,029	8.64	−0.12
	*qGR-6-2*	4P-JP13	6	77.70	bin152	3.72	76.21–78.83	17,584,761–19,743,620	6.56	−0.10
	*qGR-8-1*	4P-JP14	8	36.31	bin78	8.76	35.46–37.13	7,532,718–8,043,872	16.34	−0.16
		4P-JP13	8	36.52	bin79	8.43	35.93–37.32	7,556,192–8,184,196	16.00	−0.16
	*qGR-8-2*	NP-JP13	8	39.75	BIN113	14.21	38.95–41.04	9,306,258–10,080,193	17.74	−0.14
		NP-JP14	8	39.11	BIN111	25.14	38.52–40.54	9,227,835–10,010,956	30.58	−0.20
		4P-JP14	8	43.93	bin94	5.33	43.58–45.74	9,303,841–10,023,989	10.42	−0.13
		4P-JP13	8	44.67	bin96	6.42	42.94–46.42	9,237,682–10,467,766	12.57	−0.14
	*qGR-8-3*	NP-JP13	8	44.91	BIN130	5.47	44.21–46.87	11,021,010–11,778,831	7.28	−0.09
		NP-JP14	8	45.54	BIN131	19.73	44.86–46.75	11,195,892–11,749,684	25.00	−0.18
	*qGR-17-1*	4P-JP13	17	19.62	bin45	3.85	17.94–21.62	4,427,107–5,762,019	6.82	−0.11
	*qGR-20-1*	NP-JP13	20	4.47	BIN9	4.21	3.93–5.34	658,215–940,529	5.10	−0.08
		NP-JP13	20	7.42	BIN13	4.00	4.71–9.21	753,951–1,176,348	4.93	−0.08
NSR	*qNSR-6-1*	4P-JP14	6	26.57	bin55	4.58	23.93–26.94	5,651,702–6,555,541	7.85	−0.12
	*qNSR-6-2*	4P-JP13	6	77.93	bin153	3.92	76.51–78.72	17,641,203–19,524,807	6.42	−0.11
	*qNSR-8-1*	4P-JP13	8	36.32	bin78	11.61	35.76–37.43	7,622,922–8,184,196	21.86	−0.20
		4P-JP14	8	36.59	bin79	8.43	36.00–37.16	7,589,218–8,043,872	15.73	−0.17
	*qNSR-8-2*	NP-JP13	8	40.72	BIN115	17.54	39.11–41.52	9,392,783–10,266,569	22.00	−0.18
		NP-JP14	8	39.84	BIN113	23.56	38.43–40.67	9,211,415–10,021,207	28.93	−0.24
	*qNSR-8-3*	NP-JP13	8	44.97	BIN130	6.45	44.57–46.68	11,108,451–11,720,537	8.85	−0.11
		NP-JP14	8	45.53	BIN131	17.67	44.54–46.75	11,108,451–11,749,684	22.81	−0.21
	*qNSR-15-1*	NP-JP13	15	89.84	BIN267	5.54	89.49–90.83	48,116,968–48,437,924	6.18	−0.11
	*qNSR-15-2*	NP-JP13	15	95.40	BIN286	4.73	94.92–96.48	49,313,326–49,682,913	5.20	−0.10
EC	*qEC-1-1*	NP-JP14	1	8.73	BIN17	3.83	6.72–13.42	1,512,497–2,472,280	3.53	35.43
	*qEC-3-1*	NP-JP14	3	55.54	BIN164	4.91	55.27–58.21	36,971,415–37,823,327	4.94	41.54
	*qEC-6-1*	4P-JP14	6	77.70	bin152	3.74	76.64–78.75	17,760,273–19,524,807	6.41	58.53
	*qEC-8-1*	4P-JP14	8	36.53	bin79	5.57	34.93–37.36	7,388,023–8,184,196	9.90	73.33
		4P-JP13	8	36.54	bin79	3.64	35.12–37.30	7,532,718–8,184,196	7.56	76.32
	*qEC-8-2*	NP-JP14	8	39.87	BIN113	18.86	39.28–40.56	9,425,731–10,001,582	21.12	86.7
		NP-JP13	8	39.84	BIN113	15.00	38.74–40.73	9,271,542–10,080,193	17.57	63.92
		4P-JP14	8	46.51	bin101	3.56	44.93–46.65	9,795,796–10,560,760	6.63	59.54
	*qEC-8-3*	NP-JP14	8	49.37	BIN142	8.87	48.15–49.62	11,901,031–12,58,5965	10.70	62.70
	*qEC-15-1*	NP-JP13	15	89.85	BIN267	4.49	88.54–90.80	47,801,654–48,437,924	4.74	32.99
	*qEC-15-2*	NP-JP13	15	94.90	BIN284	4.32	93.47–96.84	48,997,333–49,682,913	4.61	32.50
	*qEC-17-1*	NP-JP14	17	44.95	BIN122	3.74	42.21–47.19	9,504,436–10,655,346	3.92	37.10
		NP-JP13	17	44.92	BIN122	4.60	42.19–47.14	9,504,436–10,655,346	5.00	33.93
	*qEC-19-1*	NP-JP14	19	112.47	BIN282	4.37	110.32–114.73	48,117,411–48,831,958	4.36	39.88

^a^ Pop-Env two populations, NP and 4P stand for two populations NJIRNP and NJIR4P. JP13 and JP14 stand for two environments Jiangpu 2013, Jiangpu 2014, respectively. QTLs detected in different environments within same, adjacent, or overlapping marker intervals were designated as the same QTL. ^b^ Chromosome Chromosome. ^c^ LOD thresholds was 3.5. ^d^ 1-LOD support confidence intervals (confidence intervals length). ^e^ Phenotypic variance (%) explained by the QTL. ^f^ The additive effect represents the genetic effects of the resistant allele contrasted with the susceptible allele of NN86-4 and NN493-1. BIN and bin markers represent the markers of different linkage maps of NJIRNP and NJIR4P, respectively.

**Table 5:** Additive QTL and interaction effects between QTL and environment for GR, NSR, and EC detected by MCIM in NJIRNP and NJIR4P populations.

Trait	QTL	Population ^a^	Chromosome ^b^	Peak Position Marker	Position (cM)	Confidence Interval (cM)	Physical Interval(bp)	A ^c^	*h*^2^ (A) (%) ^d^	*h*^2^ (AE)(%) ^e^	CIM ^f^
GR	*qGR-1-1*	4P	1	bin185	83.20	82.63–84.07	51,148,304–51,446,046	−0.07 ***	3.87	0	
	*qGR-6-1*	4P	6	bin56	27.91	26.04–28.92	6,265,471–6,843,637	−0.10 ***	6.35	0	1
	*qGR-6-2*	4P	6	bin152	77.76	76.11–78.71	17,641,203–19,524,807	−0.01 ***	6.31	0	1
	*qGR-8-1*	4P	8	bin97	44.89	43.35–46.47	9,303,841–10,467,766	−0.17 ***	20.60	0	1
		NP	8	BIN115	40.72	38.95–42.32	9,306,258–1,0467,766	−0.16 ***	20.42	0	1
	*qGR-8-3*	NP	8	BIN133	43.96	43.17–44.82	11,471,164–11,623,494	−0.05 ***	2.37	1.73	1
NSR	*qNSR-1-1*	4P	1	bin185	83.23	82.63–84.09	51,148,304–5,1446,046	−0.09 ***	3.64	0	
	*qNSR-6-1*	4P	6	bin56	27.96	25.72–30.40	6,093,115–7,371,337	−0.10 ***	5.62	0	1
	*qNSR-6-2*	4P	6	bin152	77.72	76.19–78.72	17,641,203–19,524,807	−0.09 ***	5.15	0	1
	*qNSR-8-1*	4P	8	bin96	44.43	43.35–46.47	9,451,162–10,269,314	−0.21 ***	24.00	0	1
		NP	8	BIN114	40.07	38.80–42.48	9,211,416–10,560,760	−0.23 ***	30.15	0.39	1
EC	*qEC-3-1*	NP	3	BIN165	55.86	55.21–56.78	36,971,415–37,521,106	37.78 ***	5.00	0.22	1
	*qEC-4-1*	4P	4	bin67	33.24	32.26–35.62	6,580,888–7,173,324	55.34 ***	4.72	0.14	
	*qEC-6-1*	4P	6	bin151	77.45	74.93–78.56	17,317,179–19,524,807	64.54 ***	6.43	0	1
	*qEC-6-2*	4P	6	bin36	17.71	15.74–19.12	3,558,370–4,842,348	39.13 ***	2.46	0	
	*qEC-8-1*	4P	8	bin79	36.50	35.81–37.39	7,589,218–8,184,196	68.41 ***	7.21	0	1
	*qEC-8-2*	NP	8	BIN113	39.83	39.48–40.11	9,392,783–10,021,207	80.92 ***	23.07	0.93	1
	*qEC-17-1*	NP	17	BIN122	43.96	38.93–44.90	9,162,497–10,335,777	31.18 ***	3.43	0	1
	*qEC-19-1*	NP	19	BIN234	90.21	88.54–90.75	44,450,022–45,202,579	24.06 ***	2.00	0	
	*qEC-19-2*	NP	19	BIN297	117.94	115.05–119.23	48,831,959–50,160,289	25.77 ***	2.35	0	

^a^ Pop-Env two populations, NP and 4P stand for two populations NJIRNP and NJIR4P. ^b^ Chromosome. ^c^ Additive effect: positive values indicate that PI342618B contributed the alleles for an increase in the trait value. ^d^ Phenotypic variance explained by additive QTL. ^e^ Phenotypic variance explained by additive QTL × environment interaction effect. ^f^ 1 indicate this QTL can be detected via CIM. *** *p* < 0.001.

**Table 6:** Epistatic QTL pairs for NSR and EC detected by MCIM in the NJIRNP and NJIR4P populations.

Population	Pair	QTL	Chromosome	Peak Position Marker	Position (cM)	Confidence Interval (cM)	AA ^a^	*h*^2^ (AA) (%) ^b^	*h*^2^ (AAE) (%) ^c^
NJIRNP									
	1	*qEC-9-1*	9	BIN19	7.72	6.92–9.26	−31.20 ***	3.42	0.0
		*qEC-20-1*	20	BIN245	98.11	97.13–100.20			
NJIR4P									
	2	*qNSR-3-1*	3	bin138	60.14	58.82–61.23	−0.09 ***	5.03	1.24
		*qNSR-9-1*	9	bin95	44.92	43.35–46.31			
	3	*qEC-4-1*	4	bin67	33.21	32.25–35.67	45.75 ***	3.20	0.52
		*qEC-8-1*	8	bin79	36.50	35.83–37.34			
	4	*qEC-3-1*	3	bin93	38.54	37.41–40.71	40.15 ***	2.54	0.06
		*qEC-15-1*	15	bin63	28.27	23.56–29.26			

^a^ Epistatic effects, a positive value indicates that the parental two-locus genotypes have a positive effect, otherwise the recombinants have a negative effect. ^b^ Phenotypic variance explained by epistatic QTL pair. ^c^ Phenotypic variance explained by epistatic QTL pair × environment interaction effect. *** *p* < 0.001.

**Table 7:** QTL hotspots detected by CIM in NJIRNP and NJIR4P populations in two different environments.

QTL Hotspot	Physical Range (bp)	QTL	Population	*R*^2^ (%)
6-1	5,651,702–7,896,029	*qGR-6-1*	4P	8.6
		*qNSR-6-1*		7.8
6-2	17,317,179–19,743,620	*qGR-6-2*	4P	6.5
		*qNSR-6-2*		6.4
		*qEC-6-1*		6.4
8-1	7,388,023–8,200,407	*qGR-8-1*	4P	16.0–16.3
		*qNSR-8-1*		15.7–21.8
		*qEC-8-1*		7.5–9.9
8-2	9,211,415–10,560,760	*qGR-8-2*	4P&NP	10.4–30.5
		*qNSR-8-2*	NP	22.0–28.9
		*qEC-8-2*	4P&NP	17.5–21.1
8-3	11,021,010–11,778,831	*qGR-8-3*	NP	7.2–25.0
		*qNSR-8-3*		8.8–22.8

NP and 4P represent two populations NJIRNP and NJIR4P, respectively.

**Table 8:** Effects of seed coat color on GR, NSR, and EC associated with seed-flooding tolerance in NJIRNP and NJIR4P populations in JP13 and JP14 environments.

Population	Env.	Trait	Effect of Seed Coat Color		
			Yellow	Pigmented ^a^	
				Black	Other Pigmented Colors
NJRINP	JP13	GR	0.59	0.93	0.68
		NSR	0.49	0.88	0.59
		EC (us/mL)	281.6	108.0	223.1
	JP14	GR	0.49	0.93	0.60
		NSR	0.41	0.86	0.52
		EC (us/mL)	330.3	96.2	257.1
NJRI4P	JP13	GR	0.35	0.90	0.49
		NSR	0.23	0.81	0.41
		EC (us/mL)	514.3	144.8	388.2
	JP14	GR	0.34	0.91	0.59
		NSR	0.24	0.84	0.50
		EC (us/mL)	466.9	111.8	319.0

^a^ pigmented seed-coat including black, brown, green and other hybrid colors.

## Data Availability

Data are contained within the article or Supplementary Materials.

## Supplementary Materials

The following supporting information can be downloaded at: https://www.mdpi.com/article/10.3390/plants12122266/s1, Table S1. The genetic and physical position of all bin markers of NJRINP and NJRI4P populations; Table S2. Primers used in this study; Table S3. ANOVA of GR, NSR, and EC in two RIL populations; Table S4. Analysis of nucleotide mutation position of *CHS1*, *CHS3*, and *CHS4*; Table S5. Functional annotation of 10 candidate genes associated with seed-flooding tolerance located in the QTL hotspots on Chromosome 8; Figure S1. Multiple sequence alignment describing the base sequence and amino acids sequence of *CHS1*, *CHS3*, and *CHS4* in P1(PI342618B), P2(NN86-4), and P3(NN493-1); Figure S2. Expression analysis of candidate genes in the QTL hotspot (8-2) region related to seed-flooding tolerance; Figure S3. Multiple sequence alignment describing the base sequence and amino acids sequence of *Glyma.08G122400* in P1(PI342618B), P2(NN86-4), and P3(NN493-1).

## References

[1] Boyer J.S. (1982). Plant productivity and environment. Science.

[2] Ellis M.H., Setter T.L. (1999). Hypoxia induces anoxia tolerance in completely submerged rice seedlings. J. Plant Physiol..

[3] Wu C., Zeng A., Chen P., Hummer W., Mokua J., Shannon J.G., Nguyen H.T. (2017). Evaluation and development of flood-tolerant soybean cultivars. Plant Breed..

[4] Hou F.F., Thseng F.S. (1991). Studies on the flooding tolerance of soybean seed: Varietal differences. Euphytica.

[5] Githiri S.M., Watanabe S., Harada K., Takahashi R. (2006). QTL analysis of flooding tolerance in soybean at an early vegetative growth stage. Plant Breed..

[6] Jackson M.B., Ishizawa K., Ito O. (2009). Evolution and mechanisms of plant tolerance to flooding stress. Ann. Bot..

[7] Nguyen V.T., Vuong T.D., VanToai T., Lee J.D., Wu X., Mian M.A.R., Dorrance A.E., Shannon J.G., Nguyen H.T. (2012). Mapping of quantitative trait loci associated with resistance to *Phytophthora sojae* and flooding tolerance in soybean. Crop Sci..

[8] Oosterhuis D.M., Scott H.D., Hampton R.E., Wullschleger S.D. (1990). Physiological responses of two soybean [*Glycine max* (L.) Merr.] cultivars to short-term flooding. Environ. Exp. Bot..

[9] Vantoai T., Thi T., Hoa C., Thi N., Hue N., Nguyen H., Grover Shannon J., Rahman M. (2010). Flooding tolerance of soybean [*Glycine max* (L.) Merr.] germplasm from southeast asia under field and screen-house environments. Open Agric. J..

[10] Rhine M.D., Stevens G., Shannon G., Wrather A., Sleper D. (2010). Yield and nutritional responses to waterlogging of soybean cultivars. Irrig. Sci..

[11] Sullivan M., VanToai T., Fausey N., Beuerlein J., Parkinson R., Soboyejo A. (2001). Evaluating on-farm flooding impacts on soybean. Crop Sci..

[12] VanToai T.T., St. Martin S.K., Chase K., Boru G., Schnipke V., Schmitthenner A.F., Lark K.G. (2001). Identification of a QTL associated with tolerance of soybean to soil waterlogging. Crop Sci..

[13] Cornelious B., Chen P., Chen Y., De Leon N., Shannon J.G., Wang D. (2005). Identification of QTLs underlying water-logging tolerance in soybean. Mol. Breed..

[14] Zhang J., McDonald S.C., Wu C.J., Ingwers M.W., Abdel-Haleem H., Chen P., Li Z. (2022). Quantitative trait loci underlying flooding tolerance in soybean (*Glycine max* [L.] Merr.). Plant Breed..

[15] Dhungana S.K., Kim H.S., Kang B.k., Seo J.H., Kim H.T., Shin S.O., Park C.H., Kwak D.Y., Morris B. (2020). Quantitative trait loci mapping for flooding tolerance at an early growth stage of soybean recombinant inbred line population. Plant Breed..

[16] Dhungana S.K., Kim H.S., Kang B.k., Seo J.H., Kim H.T., Shin S.O., Oh J.H., Baek I.Y. (2021). Identification of QTL for Tolerance to Flooding Stress at Seedling Stage of Soybean (*Glycine max* [L.] Merr.). Agronomy.

[17] Van N.L., Takahashi R., Githiri S.M., Rodriguez T.O., Tsutsumi N., Kajihara S., Sayama T., Ishimoto M., Harada K., Suematsu K. (2017). Mapping quantitative trait loci for root development under hypoxia conditions in soybean (*Glycine max* L. Merr.). Theor. Appl. Genet..

[18] Ye H., Song L., Chen H., Valliyodan B., Cheng P., Ali L., Vuong T., Wu C., Orlowski J., Buckley B. (2018). A major natural genetic variation associated with root system architecture and plasticity improves waterlogging tolerance and yield in soybean. Plant Cell Environ..

[19] Hou F.F., Thseng F.S. (1992). Studies on the screening technique for pre-germination flooding tolerance in soybean. Jpn. J. Crop Sci..

[20] Sayama T., Nakazaki T., Ishikawa G., Yagasaki K., Yamada N., Hirota N., Hirata K., Yoshikawa T., Saito H., Teraishi M. (2009). QTL analysis of seed-flooding tolerance in soybean (*Glycine max* [L.] Merr.). Plant Sci..

[21] Ali J., Xing G.N., He J.B., Zhao T.J., Gai J.Y. (2020). Detecting the QTL-allele system controlling seed-flooding tolerance in a nested association mapping population of soybean. Crop J..

[22] Wu C., Mozzoni L.A., Moseley D., Hummer W., Ye H., Chen P., Shannon G., Henry T.N. (2020). Genome-wide association mapping of flooding tolerance in soybean. Mol. Breed..

[23] Yu Z.P., Chang F.G., Lv W.H., Sharmin R.A., Wang Z.L., Kong J.J., Bhat J.A., Zhao T.J. (2019). Identification of QTN and Candidate Gene for Seed-flooding Tolerance in Soybean [*Glycine max* (L.) Merr.] using Genome-Wide Association Study (GWAS). Genes.

[24] Duke S.H., Kakefuda G., Henson C.A., Loeffler N.L., Van Hulle N.M. (1986). Role of the testa epidermis in the leakage of intracellular substance from imbibing soybean seeds and implications for seedling survival. Physiol. Plant..

[25] Matthews S., Powell A.A. (2006). Electrical conductivity vigour test: Physiological basis and use. Seed Test. Int..

[26] Mirdad Z., Powell A.A., Matthews S. (2006). Prediction of germination in artificially aged seeds of *Brassica* spp. using the bulk conductivity test. Seed Sci. Technol..

[27] Demir I., Cebeci C., Guloksuz T. (2012). Electrical conductivity measurement to predict germination of commercially available radish seed lots. Seed Sci. Technol..

[28] Sharmin R.A., Karikari B., Chang F.G., Al A.G.M., Bhuiyan M.R., Hina A., Lv W.H., Zhang C.T., Begum N., Zhao T.J. (2021). Genome-wide association study uncovers major genetic loci associated with seed flooding tolerance in soybean. BMC Plant Biol..

[29] Nichols D.M., Wang L.Z., Pei Y.L., Glover K., Diers B.W. (2007). Variability among Chinese *Glycine soja* and Chinese and North American soybean genotypes. Crop Sci..

[30] Lee J.D., Yu J.K., Hwang Y.H., Blake S., So Y.S., Lee G.J., Nguyen H.T., Shannon J.G. (2008). Genetic diversity of wild soybean (*Glycine soja* Sieb. and Zucc.) accessions from South Korea and other countries. Crop Sci..

[31] Pantalone V.R., Rebetzke G.J., Burton J.W., Wilson R.F. (1997). Genetic regulation of linolenic acid concentration in wild soybean *Glycine soja* accessions. J. Am. Oil Chem. Soc..

[32] Sebolt A.M., Shoemaker R.C., Diers B.W. (2000). Analysis of a quantitative trait locus allele from wild soybean that increases seed protein concentration in soybean research. Crop Sci..

[33] Chen Y., Chen P., De los Reyes B.G. (2006). Differential responses of the cultivated and wild species of soybean to dehydration stress. Crop Sci..

[34] Winter S.M.J., Shelp B.J., Anderson T.R., Welacky T.W., Rajcan I. (2007). QTL associated with horizontal resistance to soybean cyst nematode in *Glycine soja* PI464925B. Theor. Appl. Genet..

[35] Nguyen H.T., Shannon J.G., Lee J.D., Vuong T.D. (2009). Inheritance of salt tolerance in wild soybean (*Glycine soja* Sieb. and Zucc.) accession PI483463. J. Hered..

[36] Zhu D., Cai H., Luo X., Bai X., Deyholos M.K., Chen Q., Chen C., Ji W., Zhu Y. (2012). Over-expression of a novel JAZ family gene from *Glycine soja*, increases salt and alkali stress tolerance. Biochem. Biophy. Res. Commun..

[37] Valliyodan B., Ye H., Song L., Murphy M., Shannon J.G., Nguyen H.T. (2017). Genetic diversity and genomic strategies for improving drought and waterlogging tolerance in soybeans. J. Exp. Bot..

[38] Sharmin R.A., Bhuiyan M.R., Lv W.H., Yu Z.P., Chang F.G., Kong J.J., Bhat J.A., Zhao T.J. (2020). RNA-Seq based transcriptomic analysis revealed genes associated with seed-flooding tolerance in wild soybean (*Glycine soja* Sieb. & Zucc.). Environ. Exp. Bot..

[39] Jaffer A.M., Yu Z.P. (2018). Establishment of evaluation procedure for soybean seed-flooding tolerance and its application to screening for tolerant germplasm sources. Legume Res..

[40] Base S. (2011). 9.3 Procedures Guide: Statistical Procedures.

[41] Nyquist W.E., Baker R.J. (1991). Estimation of heritability and prediction of selection response in plant populations. Crit. Rev. Plant Sci..

[42] Wang W.B., Liu M.F., Wang Y.Q., Li X., Cheng S., Shu L., Yu Z., Kong J.J., Zhao T.J., Gai J.Y. (2016). Characterizing two inter-specific bin maps for the exploration of the QTLs/genes that confer three soybean evolutionary traits. Front. Plant Sci..

[43] Wang S., Basten C., Zeng Z. (2010). Windows QTL Cartographer 2.5.

[44] Hu C., Hu H., Yang J., Zhu J., Yu R., Ye X., Xia Z. (2008). QTL Network: Mapping and visualizing genetic architecture of complex traits in experimental populations. Bioinformatics.

[45] Churchill G.A., Doerge R.W. (1994). Empirical threshold values for quantitative trait mapping. Genetics.

[46] Xu H., Zhu J. (2012). Statistical approaches in QTL mapping and molecular breeding for complex traits. Chin. Sci. Bull..

[47] Todd J.J., Vodkin L.O. (1996). Duplications that suppress and deletions that restore expression from a chalcone synthase multigene family. Plant Cell.

[48] Mancuso S., Shabala S. (2010). Waterlogging Signalling and Tolerance in Plants.

[49] Tamang B.G., Magliozzi J.O., Maroof M.A.S., Fukao T. (2014). Physiological and transcriptomic characterization of submergence and reoxygenation responses in soybean seedlings. Plant Cell Environ..

[50] Sakuma Y., Liu Q., Dubouzet J.G., Abe H., Shinozaki K., Yamaguchi S.K. (2002). DNA-Binding specificity of the ERF/AP2 domain of Arabidopsis DREBs, transcription factors involved in dehydration- and cold-inducible gene expression. Biochem. Biophy. Res. Commun..

[51] Attila K., Dongxue L., Arief I., Nguyen C.D.T., Sureeporn N., Mark K., Gresshoff P.M. (2007). Agrobacterium rhizogenes-mediated transformation of soybean to study root biology. Nat. Protoc..

[52] Zhao T.J., Aleem M., Sharmin R.A. (2017). Adaptation to Water Stress in Soybean: Morphology to Genetics. Plant, Abiotic Stress and Responses to Climate Change.

[53] Wu C., Mozzoni L., Hummer W., Chen P., Shannon J.G., Ye H., Nguyen H.T., Kaur G., Orlowski J., Carter T. (2018). Advances in flood-tolerant varieties of soybean. Achieving Sustainable Cultivation of Soybeans.

[54] Rizal G., Karki S. (2011). Alcohol dehydrogenase (ADH) activity in soybean (*Glycine max* [L.] Merr.) under flooding stress. Electron. J. Plant Breed..

[55] Allard R.W. (1996). Genetic basis of the evolution of adaptedness in plants. Euphytica.

[56] Carlborg Ö., Haley C.S. (2004). Epistasis: Too often neglected in complex trait studies?. Nat. Rev. Genet..

[57] Shan D.P., Zhao Q.I., Qiu H.M., Shan C.Y., Liu C.Y., Guo H.U., Chen Q.S. (2008). Epistatic effects of QTLs and QE interaction effects on oil content in soybean. Acta Agron. Sin..

[58] Cao Y.C., Li S.G., He X.H., Chang F.G., Kong J.J., Gai J.Y., Zhao T.J. (2017). Mapping QTLs for plant height and flowering time in a Chinese summer planting soybean RIL population. Euphytica.

[59] Clough S.J., Tuteja J., Li M., Marek L.F., Shoemaker R.C., Vodkin L.O. (2004). Features of a 103-kb gene-rich region in soybean include an inverted perfect repeat cluster of CHS genes comprising the *I* locus. Genome.

[60] Xu K.N., Xu X., Fukao T., Canlas P., Maghirang-Rodriguez R., Heuer S., Ismail A.M., Bailey-Serres J., Ronald P.C., Mackill D.J. (2006). Sub1A is an ethylene-response-factor-like gene that confers submergence tolerance to rice. Nature.

[61] Hattori Y., Nagai K., Furukawa S., Song X.J., Kawano R., Sakakibara H., Wu J.Z., Matsumoto T., Yoshimura A., Kitano H. (2009). The ethylene response factors *SNORKEL1* and *SNORKEL2* allow rice to adapt to deep water. Nature.

[62] Bui L.T., Giuntoli B., Kosmacz M., Parlanti S., Licausi F. (2015). Constitutively expressed ERF-VII transcription factors redundantly activate the core anaerobic response in Arabidopsis thaliana. Plant Sci..

