# Supplementary material for: Genetic Dissection of Extreme Seed-Flooding Tolerance in a Wild Soybean PI342618B by Linkage Mapping and Candidate Gene Analysis
Source: Plants (Basel). 2023 Jun 10;12(12):2266. doi: 10.3390/plants12122266 (PMC10302228; doi:10.3390/plants12122266)
Supplement: Supplementary file 1 [file plants-12-02266-s001.zip › plants-2279748-supplementary.pdf]

Supplementary Table S1

Primers used in this study

Gene	Forward (5'-3')	Reverse (5'-3')	Purpose
CHS1-Glyma.08G109400 cds	ATGGTGAGTGTTGAAGAGATTC	TCAGAGAGTGACACTGCGGAGC	gene cloning
CHS3-Glyma.08G109300 cds	ATGGTCAGTGTTGAAGAGATCCGTA	TTAGACAGTGACACTGCGGAGCACA	gene cloning
CHS4-Glyma.08G110700 cds	ATGGTGAGTGTTGAAGAGATTCGTA	TTAGACAGTGACACTGCGGAGTACA	gene cloning
Glyma.08g122400 cds	ATGAATGCTAGAAGCTGGCA	CTAGATGTAATTTGCTAGTTGA	gene cloning
Glyma.08g125100 cds	ATGGAGGTGACCAACCTTGT	CTAGAAATGGGAAGGATGGA	gene cloning
Glyma.08G137600 cds	ATGGGAACTGCTATAGACATGT	TCATATAGCTTCCCAATCAATC	gene cloning
Glyma.08g145300 cds	ATGATGAAGAACGATAGAGA	TCAGGCAGAAGAAGAGGAGC	gene cloning
Glyma.08G149200 cds	ATGGCAGAGTCTCAGAAACAAC	TTAGGCATAATGTATATTGAGGGG	gene cloning
qPCR-*Glyma.08g125600*	CGCATGCAAGAAATAGGTTC	TGTAATTGACTGCACACATT	qRT-PCR expression analysis
qPCR-*Glyma.08g119500*	TGGTGCATTTTCAGAACTCA	TCACGTTTATCTGATACCCT	qRT-PCR expression analysis
qPCR-*Glyma.08g119600*	AACTTAACTCACAGCTCGTA	GGGTATCAAAGTTTGAGATT	qRT-PCR expression analysis
qPCR-*Glyma.08g122400*	ATAATCCGAACACTGTCCGC	GTTGGTGGTGGCTGATTAGT	qRT-PCR expression analysis
qPCR-*Glyma.08g125000*	TTCCTGGAACAAGGTTTCAT	CATTTCGGTCATAAACCTTC	qRT-PCR expression analysis
qPCR-*Glyma.08g125100*	CTCAGAAACTACTTGCCAAA	AAATTTTAAGGAAAGCTTTG	qRT-PCR expression analysis
qPCR-*Glyma.08g128600*	CAGAAAGTTTGAGGACTTGT	ATTGGCATGTGGCTGAAATT	qRT-PCR expression analysis
qPCR-*Glyma.08g145300*	TCATGGTGGCAACTCTGTTCTC	CTCCTCGCTTCTTCTTCCTCCT	qRT-PCR expression analysis
qPCR-*Glyma.08G137600*	TCTAAGACACCACGGAGCCTTC	TTTGCCACGTCAGACTCGACCT	qRT-PCR expression analysis
qPCR-*Glyma.08G149200*	TTGGAGAGGATTCAGAACCCGA	CCCATTTTCTCTGCCTAACACC	qRT-PCR expression analysis
qRT-*Actin11*	ATGACCCAAATCATGTTTGAGACC	TCAGTTAGATCACGACCAGCGA	qRT-PCR expression analysis
GmSFT1(*Glyma.08G137600*)	AGCCACCATGCTCGAGATGGGAAC TGCTATAGACATGTAC	TGCTCACCATCTCGAGTATAGCTTC CCAATCAATCTCCAC	vector construction

Table S2

ANOVA of GR, NSR and EC in two RIL populations.

Population	Trait	Source	DF[a]	MS[b]	*F*[c]
NJRINP	GR	Genotype	280	0.37	2.43***
		Env	1	1.87	12.32***
		Block	1	0.00	0.00
		Gen × Env	265	0.17	1.09**
		Error	485	0.15	
	NSR	Genotype	280	0.17	12.05***
		Env	1	46.23	3300.74***
		Block	1	0.07	4.83
		Gen × Env	264	0.13	9.29***
		Error	482	0.01	
	EC	Genotype	280	131017.63	23.36***
		Env	1	139454.82	24.87***
		Block	1	23624.23	4.21
		Gen × Env	269	13471.06	2.4**
		Error	541	5607.53	

Population	Trait	Source	DF[a]	MS[b]	*F*[c]
NJRI4P	GR	Genotype	160	0.43	9.08***
		Env	1	0.64	13.34***
		Block	1	0.00	0.00
		Gen × Env	159	0.10	2.01**
		Error	302	0.05	
	NSR	Genotype	160	0.44	11.23***
		Env	1	0.29	7.26**
		Block	1	0.00	0.01
		Gen × Env	158	0.09	2.37**
		Error	282	0.04	
	EC	Genotype	160	185786.52	8.69***
		Env	1	137737.31	6.44**
		Block	1	15016.54	0.70
		Gen × Env	159	37284.26	1.74**
		Error	312	21388.16	

*** $p<0.001$; ** $p<0.01$. [a] Degrees of freedom. [b] Mean square. [c] *F* value is for determining significance.

Table S3

Analysis of nucleotide mutation position of *CHS1*, *CHS3* and *CHS4*.

Gene	Position(bp)	Nucleotide variation	Amino acid variation	
			Variation way	Variation type
CHS1	396	T/C	-	S
	498	C/T		
	531	C/T		
CHS3	219	T/C		
	225	A/G		
	243	G/C		
	255	T/A		
	258	C/T		
CHS4	531	C/T		

x/y = x nucleotide change into y nucleotide, - = No variation, S = Synonymous mutations, N = Non-synonymous mutations.

Table S4

Functional annotation of 10 candidate genes associated with seed-flooding tolerance located in the QTL hotspots on Chr.08.

Gene	Function annotation
Glyma.08g119500	FASCICLIN-like arabinogalactan-protein 12
Glyma.08g119600	FASCICLIN-like arabinogalactan-protein 11
Glyma.08g122400	Carbohydrate-binding X8 domain superfamily protein
Glyma.08g125000	Cytochrome P450, family 716, subfamily A, polypeptide 1
Glyma.08g125100	Cytochrome P450, family 716, subfamily A, polypeptide 1
Glyma.08g125600	UDP-glycosyltransferase 74 F1
Glyma.08g128600	Cytochrome P450, family 716, subfamily A, polypeptide 1
Glyma.08G137600	Ethylene response factor/AP2 domain
Glyma.08g145300	Integrase-type DNA-binding superfamily protein/AP2 domain
Glyma.08G149200	Integrase-type DNA-binding superfamily protein/AP2 domain

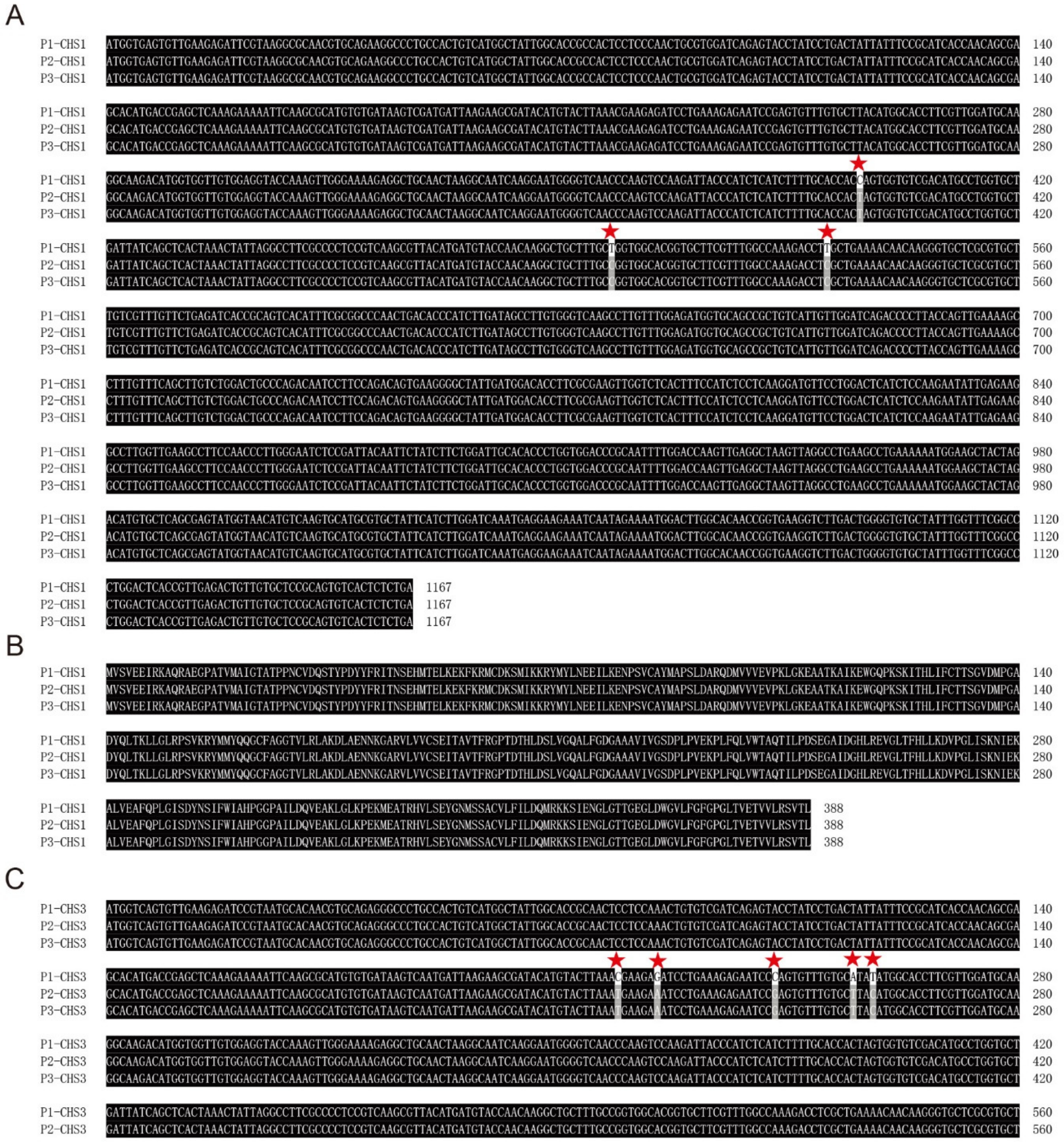

Supplementary Fig. S1. Multiple sequence alignment describing the base sequence and amino acids sequence of *CHS1*, *CHS3* and *CHS4* in P1(PI342618B), P2(NN86-4) and P3(NN493-1). (A),(C),(E) represent multiple base sequence alignment of *CHS1*, *CHS3* and *CHS4*. (B),(D),(F) represent multiple amino acids sequence alignment of *CHS1*, *CHS3* and *CHS4*.

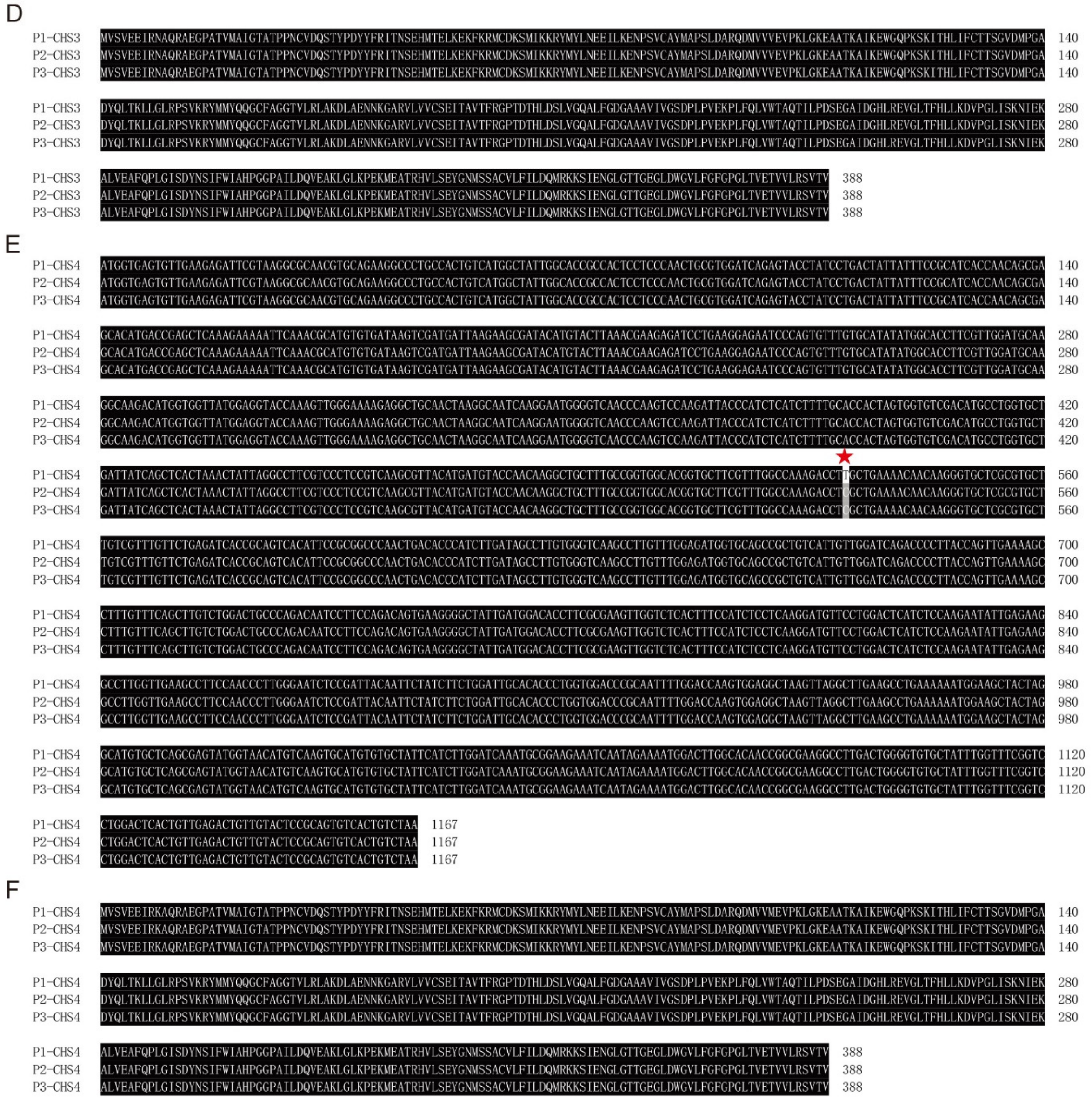

Supplementary Fig. S1. Multiple sequence alignment describing the base sequence and amino acids sequence of *CHS1*, *CHS3* and *CHS4* in P1(PI342618B), P2(NN86-4) and P3(NN493-1). (A),(C),(E) represent multiple base sequence alignment of *CHS1*, *CHS3* and *CHS4*. (B),(D),(F) represent multiple amino acids sequence alignment of *CHS1*, *CHS3* and *CHS4*.

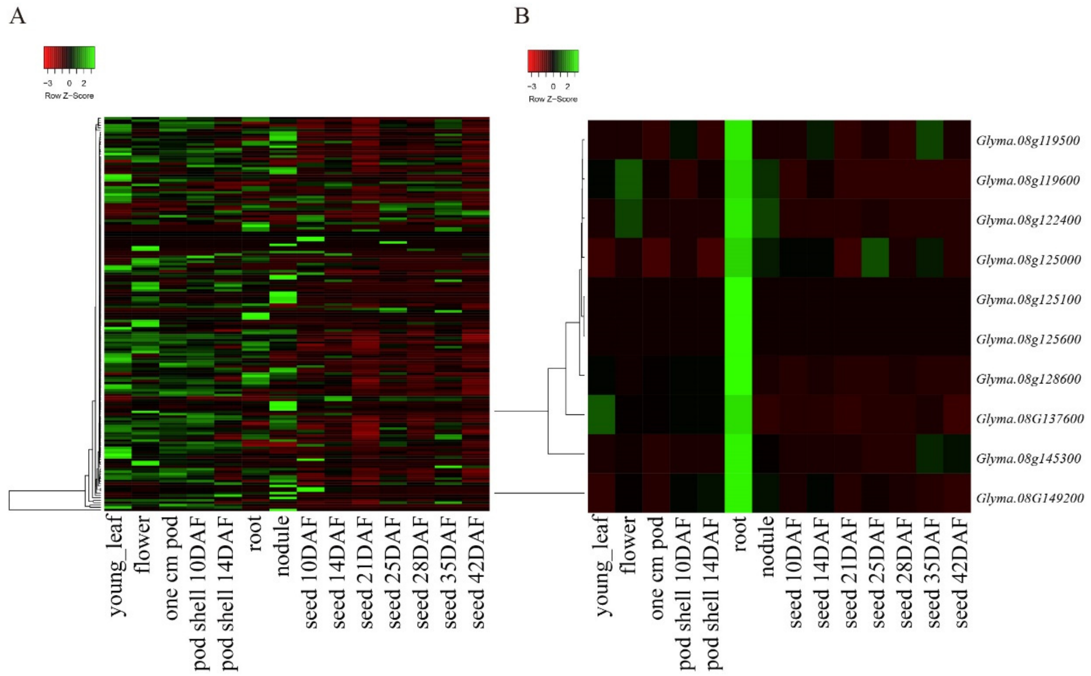

Supplementary Fig. S2. Expression analysis of candidate genes in the QTL hotspot(8-2) region related to seed-flooding tolerance. (A) Heat maps of expression levels for 164 candidate genes in different soybean tissues. (B) Heat maps of expression levels for 10 screened candidate genes associated with seed-flooding tolerance in different tissues. The RNA-seq data were were obtained from Soybase (https://www.soybase.org/soyseq/).

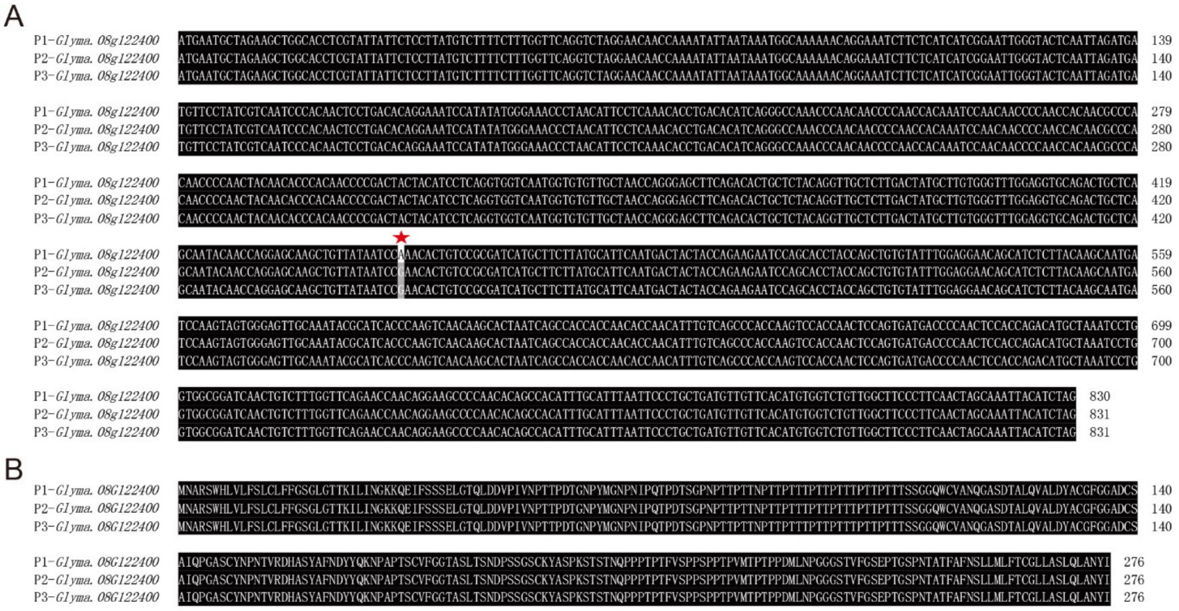

Supplementary Fig. S3. Multiple sequence alignment describing the base sequence and amino acids sequence of *Glyma.08G122400* in P1(PI342618B), P2(NN86-4) and P3(NN493-1). (A) represent multiple base sequence alignment of *Glyma.08G124400*. (B) represent multiple amino acids sequence alignment of *Glyma.08G124400*.
